# New players in the same old game: a system level *in silico* study to predict type III secretion system and effector proteins in bacterial genomes reveals common themes in T3SS mediated pathogenesis

**DOI:** 10.1186/1756-0500-6-297

**Published:** 2013-07-26

**Authors:** Vineet Sadarangani, Sunando Datta, Manonmani Arunachalam

**Affiliations:** 1 National Institute of Immunology, Aruna Asaf Ali Marg, New Delhi 110067, India; 2 Indian Institutes of Science Education and Research, Bhopal 462023, India; 3 UT Southwestern Medical Center, Department of Molecular Biology, Dallas, TX 75235, USA

**Keywords:** T3SS, Effector, Intracellular pathogen, Cytoskeletal structure

## Abstract

**Background:**

Type III secretion system (T3SS) plays an important role in virulence or symbiosis of many pathogenic or symbiotic bacteria [CHM 2:291–294, 2007; Physiology (Bethesda) 20:326–339, 2005]. T3SS acts like a tunnel between a bacterium and its host through which the bacterium injects ‘effector’ proteins into the latter [Nature 444:567–573, 2006; COSB 18:258–266, 2008]. The effectors spatially and temporally modify the host signalling pathways [FEMS Microbiol Rev 35:1100–1125, 2011; Cell Host Microbe5:571–579, 2009]. In spite its crucial role in host-pathogen interaction, the study of T3SS and the associated effectors has been limited to a few bacteria [Cell Microbiol 13:1858–1869, 2011; Nat Rev Microbiol 6:11–16, 2008; Mol Microbiol 80:1420–1438, 2011]. Before one set out to perform systematic experimental studies on an unknown set of bacteria it would be beneficial to identify the potential candidates by developing an *in silico* screening algorithm. A system level study would also be advantageous over traditional laboratory methods to extract an overriding theme for host-pathogen interaction, if any, from the vast resources of data generated by sequencing multiple bacterial genomes.

**Results:**

We have developed an *in silico* protocol in which the most conserved set of T3SS proteins was used as the query against the entire bacterial database with increasingly stringent search parameters. It enabled us to identify several uncharacterized T3SS positive bacteria. We adopted a similar strategy to predict the presence of the already known effectors in the newly identified T3SS positive bacteria. The huge resources of biochemical data [FEMS Microbiol Rev 35:1100–1125, 2011; Cell Host Microbe 5:571–579, 2009; BMC Bioinformatics 7(11):S4, 2010] on the T3SS effectors enabled us to search for the common theme in T3SS mediated pathogenesis. We identified few cellular signalling networks in the host, which are manipulated by most of the T3SS containing pathogens. We went on to look for correlation, if any, between the biological quirks of a particular class of bacteria with the effectors they harbour. We could pin point few effectors, which were enriched in certain classes of bacteria.

**Conclusion:**

The current study would open up new avenues to explore many uncharacterized T3SS positive bacteria. The experimental validation of the predictions from this study will unravel a generalized mechanism for T3SS positive bacterial infection into host cell.

## Background

Many symbiotic and pathogenic Gram -ve bacteria have evolved with sophisticated secretion machinery. These secretion systems differ in their architectures and types of the bio- molecules they secrete. There are at least 6 specialized secretion systems - Type I, Type II, Type III, Type IV, Type V and Type VI found in Gram-ve bacteria [1]. Type III secretion system is known to be specialized in injecting proteins into host cells [2,3]. T3SS plays a central role in the pathogenesis or survival in the host [4-6].

T3SS consists of a hollow proteinaceous channel that spans both inner and outer membrane of bacteria and thus facilitates the transfer of bacterial proteins to the host cell. The current knowledge on the structure of this machinery have been derived from the reconstruction-based approach in which the X-ray crystal structures of the individual proteins were fitted into a low resolution electron density mask of the entire injectisome complex [3,7,8]. The T3SS machinery is a modular system, which has a basal body spanning inner membrane, peri-membrane space and the outer membrane of the bacterium. The needle along with its tip is inserted into the basal body. Approximately 25 different proteins are required to build the needle complex [9,10]. The comparison of injectisomes from many well-studied T3SS positive bacteria revealed that the basal structure is made of ten highly conserved proteins.

The injectisomes allow transport of the proteins, known as effectors from the pathogens to host cells. The latter group of proteins have diverse biochemical activities and play pivotal role in pathogen mediated infection in plants and animals [5,11]. In many occasions, these proteins containing eukaryotic protein motifs mimic the functions of its host counterpart and thus modulate different signalling pathways [12]. So far, the studies on T3SS have been limited to very few bacteria [13-15]. Although numerous independent studies have been performed in individual pathogens, they mainly focussed on the physiological role of a subset of effectors in a given pair of pathogen and its host. The rapid increase in the number of available sequences of bacterial genomes offers opportunity to identify new potential T3SS positive bacteria. Additionally, it also provides a solid framework for carrying out system level study to extract an overriding theme for pathogenesis.

Here, we made use of the vast resources of data generated by sequencing multiple bacterial genomes to identify un-annotated T3SS positive bacteria. We then asked whether there is any relation between the natural habitat of a bacterium and its effectors. To address this we divided the entire pool of T3SS positive bacteria into different classes according to their natural habitats and set out to look for enrichment of a set of effectors in a given class. Our study identified several un-annotated T3SS positive bacteria. Interestingly, couple of them maintain very distant relations with the known classes of T3SS positive bacteria and thus likely to constitute a new family [9]. We found both habitat dependent and independent enrichment of the effectors. Certain groups of effectors were found to be present in most of the T3SS positive bacteria irrespective of the habitat. These effectors have been shown to modulate specific signalling pathways in the host. This led us to hypothesize that T3SS positive bacteria modulate certain conserved signalling pathways in the host irrespective of their habitat. Interestingly, we also observed habitat dependent enrichment of certain effectors.

## Methods

### Bacterial genome data set

The curetted sequences of the bacterial genomes or plasmids are available in NCBI database (http://ftp://ftp.ncbi.nih.gov/genomes/Bacteria/). This data set as on 14^th^ of April, 2012 contained nearly 1430 bacterial genome sequences.

### Experimentation tools

**Figure 1:**
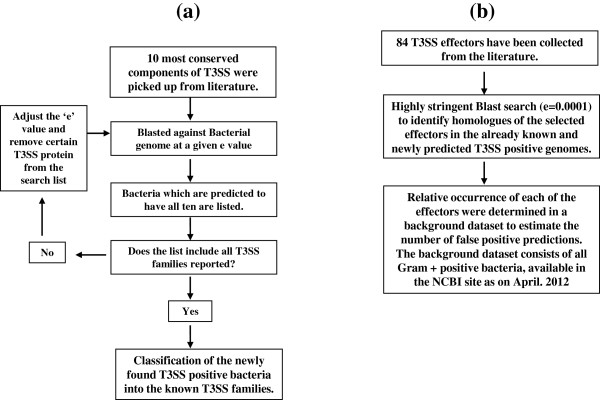
**Flow chart diagram of the methodology used for identification of T3SS positive bacteria and their effectors. 1a**: Prediction of T3SS. **1b**: Prediction of their effectors.

We have used BLAST (2.2.26 release) for searching homologues of T3SS components against the bacteria genomes [16]. We used ‘tblastn’ algorithm, which is a tool that compares a protein query sequence against a nucleotide sequence database, dynamically translated in all six reading frames (both strands). The blast searches were carried out at multiple ‘e’ values. Here ‘e’ value is a parameter that describes the number of hits one can expect to see by chance when searching database of a particular size. We carried out multiple sequence alignment (MSA) using clustalW [17] through GenomeNet bioinfomatics online tools. (http://www.genome.jp/tools/clustalw/). The same tool was then used to build a phylogram from the multiple sequence alignment (unweighted Pair Group Method with Arithmetic mean). The methodology used to predict T3SS positive genomes and their effectors are discussed in detail in the following section and shown as a flow chart in Figure 1.

## Results and discussion

### Prediction of T3SS components in bacteria

**Figure 2:**
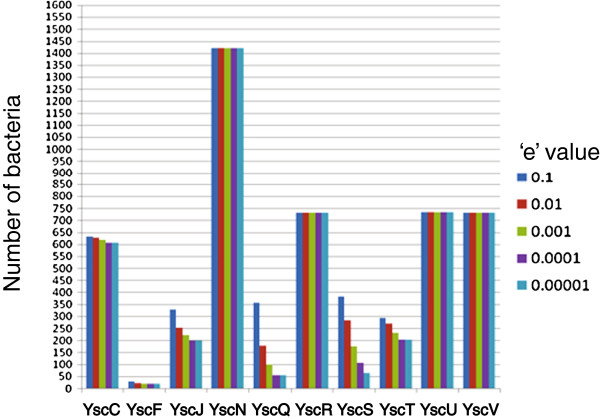
**Frequency of occurrences of individual T3SS proteins in bacterial genome.** The homologues of each of the T3SS proteins have been predicted in bacterial genomes at different ‘e’ values. The numbers are plotted as a bar diagram. The colour codes used for different ‘e’ values are shown in the figure. ‘e’ values of 0.1, 0.01, 0.001, 0.0001, 0.00001 are shown in dark blue, red, green, magenta and light blue respectively.

Over 25 proteins have been proposed to constitute a functional T3SS and 10 (YscC, YscF, YscJ, YscN, YscQ, YscR, YscS, YscT, YscU and YscV) are well conserved in most of the known T3SS positive bacteria [9]. All these proteins except YscF constitute the basal structure of the T3SS machinery. YscF constitutes the needle of the injectisome [7]. An extensive search of these 10 T3SS proteins has been carried out in bacterial genomes using decreasing ‘e’ value. Additional file 1 shows the relative occurrence of each of the ten T3SS proteins. The same result is shown as a bar diagram in Figure 2. It is clear from the Additional file 1 and Figure 2 that the T3SS proteins could be classified in four groups. The first group of proteins highlighted in the shade of cyan, which consists of YscC, YscR, YscU and YscV is present in almost 50% of the bacteria and are present at all the ‘e’ values used in this analysis. Hence they might be involved in housekeeping activities. YscC is a member of secretin family of proteins and present in many bacteria with secretion machinery [3,7,9]. YscR, YscU and YscV are thought to be present in the inner membrane of the basal body [6,9]. YscN, highlighted in red, is an ATPase, and hence expected to have homologues in every bacterium. Among 10 core components, YscN protein was the most frequently found one. It is thought to serve as the energy source for transporting effectors/translocators through the injectisome [3,9,10]. YscJ, YscS and YscT constitute the third group, highlighted in dark green that was proposed to be part of the basal body in different types of secretion systems. YscQ and YscF, highlighted in magenta represent the fourth and final group. The recent experimental evidence suggested that YscQ plays crucial role in assembly of the apparatus [10]. The rigorous search method involving increasing stringency revealed interesting properties of the T3SS proteins. YscQ and YscF were found to be most sensitive to the change in the stringency of search method. At a given ‘e’ value the presence of these two components assures the presence of the other 8 T3SS components. Thus these proteins can serve as the real indicator for the presence of T3SS in a bacterium. In contrary, failure to detect them by *in silico* methods will not rule out the presence of a T3SS in a given bacterium since at lower ‘e’ values, both the proteins were found to be absent in many of the well characterized T3SS positive bacteria. It also indicates that the functions carried out by these proteins might not require any conserved sequence motifs.

### T3SS predicted in bacterial genome

**Figure 3:**
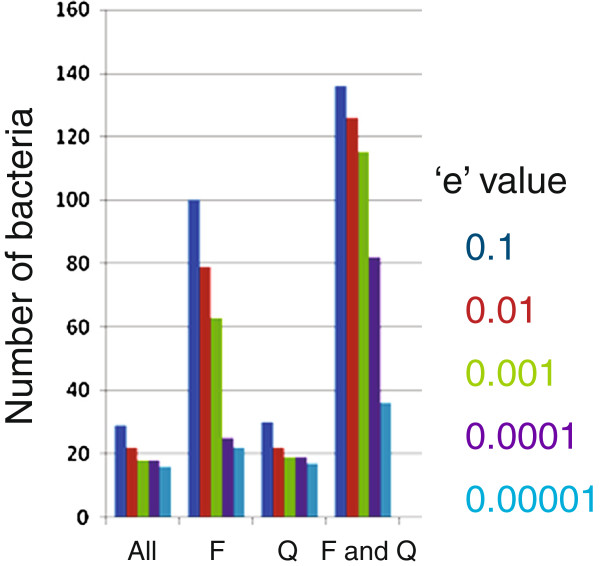
**Prediction of T3SS positive bacteria at different ‘e’ values.** The number of bacteria which were predicted to harbour ‘All’: all 10 T3SS proteins, ‘F’: all but YscF, ‘Q’: all but YscQ and ‘F & Q’: all but YscF and YscQ are plotted as a bar diagram. The colour codes used for different ‘e’ values are shown in the figure. ‘e’ values of 0.1, 0.01, 0.001, 0.0001, 0.00001 are shown in dark blue, red, green, magenta and light blue respectively.

We hypothesized that the bacteria containing all ten conserved T3SS genes are likely to harbour a functional T3SS and hence started identifying the bacteria which have all ten T3SS genes. Interestingly, some of the T3SS proteins have their homologues also present in bacterial flagella system. But, the criterion of co-incidence detection of all ten components in a given genome has made the current analysis amenable for identification of T3SS. Table 1 shows the number of such T3SS positive bacteria at each ‘e’ value. It also shows the number of bacteria which lack either of YscF and YscQ or both. The result is shown as a bar diagram in Figure 3. As expected, we could detect more number of T3SS positive bacteria by relaxing the constraint of either of these two proteins. It is evident from Figure 3 that the number of predicted T3SS positive bacteria depends modestly on the ‘e’ value but it is much more sensitive to inclusion of YscF or YscQ in the search criterion. In order to choose an appropriate value for ‘e’ value, we have used the phylogenetic data already available in the literature. Based on phylogenetic analysis of the ATPase, YscN, Cornelis *et al.* classified the T3SS positive bacteria into seven distinct families [9]. We chose the parameters for our analysis in such a way that we could predict at least one member from each family. At ‘e’ value 0.1 and with all 10 T3SS protein used for prediction, we could only detect Ysc, Hrc1, Inv-Mxi-Spa and Ssa-Esc families out of the total seven families. We could include the Hrc2 and Rhizobium family only when YscF was omitted. Upon omitting YscQ from the analysis, we could include a single member, *Shigella boydii,* which was already known to contain a functional T3SS. Upon relaxing both YscF and YscQ, we could include members from all 7 families including Chlamydia family. We performed the calculation using decreasing ‘e’ values. Figure 3 shows the number T3SS positive bacteria for each of the ‘e’ values with or without including YscF or YscQ. We found a large change in the number of the bacteria when we reduce the ‘e’ value from 0.001 to 0.0001 (Figure 2b). At a value of 0.0001 and keeping all ten proteins as the condition, we could detect only couple of families of T3SS positive bacteria. On the basis of our observation, we decided to use an ‘e’ value of 0.001 for our further analysis. In order to include at least one member from each of the families and all the experimentally verified T3SS positive bacteria, we excluded YscF, YscQ and YscS from our search criteria. Using the above criteria, we could detect 151 T3SS positive bacteria in the entire bacterial kingdom (Additional file 2: Table S1). It includes 85 unique species and 43 unique genera. 18 new bacteria have been predicted. In order to identify the family to which each of these newly identified T3SS candidates belongs, we carried out phylogenetic analysis of them based on the multiple sequence alignment of the most conserved T3SS component, YscN (Additional file 2: Table S5). As shown in Table 2 the newly identified, candidates are distributed over all seven families of T3SS system and diverse host organisms. The bacteria with unknown hosts are indicated by question marks. We also could not place *Selenomonas sputigena* and *Hahella chejuensis* into any of the known T3SS families. Interestingly, for the first time we could predict the presence of a T3SS in a bacterium which infects an amoeba. The list also includes four human pathogens which vary in the severity of the patho-physiology associated with them. Using the above search criteria, we could not include some of the members of the Chlamydia family including experimentally characterized *Chlamydia trachomatis*[18]*.* In order to include this obligatory intracellular pathogen, we ought to remove YscC, a conserved member of Secretin family from our query. The result presented here indicates that the functions carried out by these proteins could be accomplished by proteins with diverse amino acid sequences. Thus some of the T3SS components might have evolved convergently with their respective functional homologues. The phenomenon of convergent evolution has already been reported for some of the T3SS effectors which were described as ‘convergently evolved’ mimics of their eukaryotic functional homologues [11]. Systematic experimental verification of the results presented here would substantially broaden the area of T3SS research.

**Table 1:** **Number of T3SS positive bacteria at different ‘e’ values**^**a**^

**‘e’ value**	**All**	**F relaxed**	**Q relaxed**	**F & Q relaxed**
0.1	29	100	30	136
0.01	22	79	22	126
0.001	18	63	19	115
0.0001	18	25	19	82
0.00001	16	22	17	36

(a) Absolute numbers are shown in a tabular form. ‘All’: all 10 proteins were used to predict the presence of T3SS system. ‘F relaxed’: all but YscF proteins were used, ‘Q relaxed’: all but YscQ were used and ‘F & Q relaxed’: both YscF and YscQ were omitted from the prediction. Different ‘e’ values are shown in different colours.

**Table 2:** Newly identified candidates

**Name**	**Famliy**	**Host organism**
*Hahella chejuensis*	?	?
*Hyphomicrobium sp. MC1*	Ysc	?
*Variovorax paradoxus*	Hrc2/Inv-Mxi-Spa	Human
*Chelativorans BNC1, Geobacter bemidjiensis*	Rhizobiales	Plant, ?
*Dickeya dadantii, Marinomonas mediterranea, Herbaspirillum seropedicae, Ramlibacter tataouinensis, Cupriavidus taiwanensis*	Hrc1	Plant, ?, ?, ? , plant
*Acidovorax avenae, Sinorhizobium fredii,*	Hrc2	Plant, plant
*Parachlamydia acanthamoebae, Waddlia chondrophila, Simkania negevensis*	Chlamydiales	Amoeba, human, Human
*Shewanella baltica*	Ssa-Esc	Fish
*Proteus mirabilis*	Inv-Mxi-Spa	Human
*Selenomonas sputigena,*	?	Human

The table enlists the newly identified bacteria with T3SS machinery. The respective T3SS family and host for each of the candidate is shown in separate columns. ‘?’ implies unknown family or host.

### T3SS associated effectors are predicted

The T3SS effectors modulate the signalling network in the host cell to facilitate the bacterial survival and pathogenesis. Therefore, as the first step, we wanted to predict the existence of the already known effectors in the newly predicted T3SS positive bacteria. The overall methodology is presented as a flow chart in Figure 1. Briefly, we collected 84 effectors from the literature and examined their biochemical activity and physiological functions (Additional file 2: Table S2). We used the “blast” based strategy to predict their homologues in the already known and newly identified T3SS positive bacteria. The search was carried out at a very high stringency (e value = 0.0001) to minimize the chance of false positives. The result was tabulated in a matrix form (Additional file 2: Table S3) where each row represents a microbial species and columns represent the effectors. Out of the 84 effectors, 74 were found to have their homologues in multiple bacteria. 18 effectors were present in more than 10 bacteria. We assessed the enrichment of the effectors in T3SS positive bacteria by calculating the relative occurrence of an effector in the T3SS positive bacteria compared to that in the background data set. All the Gram + ve bacteria constituted the background set. 75 effectors were found to have no homologues in the background dataset. The number of homologues for each effectors in the background dataset is shown in parenthesis in the Additional file 2: Table S4. We could find quite a few effectors to be present in more than 15 different bacteria. PipB was the most abundant among all the effectors we studied. It associates with the late phagosomal or endosomal compartments [19,20]. Its putative presence in the extracellular bacteria strongly suggests that it might have other unknown functions. PipB is also highly abundant in Gram + ve dataset. It contains penta-peptide motif which is known to act as the hub for protein-protein interactions. Thus, PipB might have a general function in the bacterial world. The effectors, SspH1 and SlrP, were predicted to be present in nearly 20 bacteria. Interestingly both of them have Leucine rich domain and both possess Ubiquitin ligase activity. SspH1 is known to down regulate NF-KB regulated gene expression in the host during infection [21,22]. The physiological role of SlrP is not yet clear. It is known to interact with DnaJ in the host cell [23,24]. Like PipB, SlrP is also abundant in the background dataset indicating its involvement in T3SS independent processes. Presence of these three effectors in both animal and plant kingdom suggests for their conserved physiological function in T3SS mediated infection. We found only 10 effectors, which did not have any homologue. This latter group includes almost all the effectors from the obligatory intracellular pathogen, *Chlamydia trachomatis*.

### T3SS bacteria use diverse means to modify few unique host signalling pathways

Next, we wanted to identify the nodes of the host cellular signalling network that are frequently tuned by the T3SS effectors. A vast resource of data is already available on the effectors including a few *in silico* prediction tools [25-27]. We started with the classification of all the effectors according to their biochemical activities and physiological functions. Additional file 2: Table S2 enlists the effectors, their biochemical activities and the biological functions in the host. As depicted in different colours, we found three major classes of effectors, which have distinctly different biochemical activities. The largest group that consists of one fourth of the total number of effectors participates in cytoskeletal remodelling and thereby facilitates or prevents internalization of the microbe by the host. Some of them modulate the activity of the Rho family of GTPases while others act on the downstream target molecules. The other two major classes of effectors possess Cysteine protease and Ubiquitin ligase activities. Through a disparate set of cellular targets, they help pathogens to circumvent the host defence system. The rest of the effectors posses diverse biochemical activities including Phosphatase, Phospho-lipase, Adenylate cyclase and their cellular targets also vary in their nature but most of them are involved in protecting the microbe from the host immune response and creating a conducive milieu for their survival and growth. It is thus evident from our study that these diverse set of bacteria contain few common groups of effectors with similar biochemical or physiological activities. One of the major groups regulates Rho GTPase mediated cytoskeletal remodelling in the host. Thus, most of the T3SS positive bacteria employ diverse types of effector molecules to regulate certain common signalling pathways in the host.

### Habitat specific enrichment of T3SS effectors

The predicted set of T3SS positive bacteria represents a very diverse biological niche. It encouraged us to investigate the habitat specific enrichment of the effectors, if any. To begin with, we made a gross classification of all the T3SS positive bacteria into intra or extracellular and plant or animal groups based on their cellular location or host they colonize (Additional file 2: Table S2). This classification is solely based on literature mining and hence the bacteria without any habitat information have been excluded from this analysis. We considered only those effectors which were present in at least ten T3SS positive bacteria. We decided to associate an effector with a particular biological niche only if it was predicted to exist in multiple bacterial species which belong to the same habitat group. We identified three effectors, namely, AvrppHB, AvrPtoB and XopD (highlighted in green) that were present only in the plant bacteria. Among these, AvrppHB and XopD have Cysteine protease domain and AvrptoB has E3 Ubiquitin ligase activity. Similarly, we also found several effectors to be present only in animal bacteria (highlighted in yellow). We termed an effector animal specific only when they could be identified in three or more different species of animal bacteria and none of the plant bacteria. The animal specific effectors were found to have two major types of activities, cysteine protease and regulators of Rho GTPases. We also identified a group of effectors that were exclusively associated with intracellular bacteria like *Shigella, Salmonella, Burkholderia* and *Yersinia* (shown underlined). The group includes four effectors, BopA, SopE, IpaA and IpaC, all of which facilitates the entry or exit of the associated microbes into or from the phagosome in the host cell. The presence of any of these enriched effectors in an uncharacterized microbe might help us in identifying the biological habitat of the same.

## Conclusion

The current study predicted the presence of T3SS machinery and associated effector proteins in several uncharacterized bacterial genomes. It also indicates that the T3SS effectors from diverse types of bacteria exploit few common signaling pathways in the host. In many occasions, the bacterial effectors differ substantially in their sequences and structures from their functional homologues in the host which makes them attractive drug targets in the pharmaceutical industry. Moreover, we detected enrichment of certain effectors in specific biological niche although the underlying molecular principles are yet to be unraveled. The current study thus created a plenty of room to explore into many uncharacterized T3SS positive bacteria. To the best of our knowledge, it is the first ever attempt to unravel an underlying theme for T3SS mediated pathogenesis from the huge resource of data. Careful wet lab validation of the current predictions should be compulsory before carrying out an experimental system level investigation on T3SS mediated pathogenesis.

## Abbreviations

T3SS: Type III secretion system.

## Competing interests

The authors declare that they have no competing interests.

## Authors’ contributions

SD and MA initiated the study. MA carried out the initial scripting for carrying out T3SS prediction. VS carried out the prediction of T3SS. SD carried out the effector analysis. SD and VS drafted and revised the manuscript. All authors approved the final version of the manuscript.

## Supplementary Material

Additional file 1 Relative occurrences of T3SS proteins in bacterial kingdom. Click here for file

Additional file 2: Figure S1 Phylogram of the 85 unique T3SS positive species based on the multiple sequence alignment of YscN, the most conserved T3SS component. **Table S1.** List of T3SS positive bacteria predicted in this study. It includes 152 unique serovars, 85 unique genera and 43 unique species. The newly identified bacteria are shown in gray shade. **Table S2.** Biochemical and physiological functions of the effectors. Four major classes are shown in different background colours. Effectors, which regulate the Rho GTPase, its downstream molecules or actin polymerization/de-polirization are shown with yellow background. Effectors with Cysteine protease activity are shown with cyan background. Effectors which modulate host cell function by ubiqutination of host factors are highlighted in magenta. Phosphatases are shown in dark green background. ‘NA’ implies the non-availability of data for a given effector. **Table S3.** Effector matrix for 152 T3SS positive bacteria. ‘1’ implies presence of a particular effector in a given bacterium and ‘0’ implies its absence. The plant specific effectors are shown in green background, whereas the animal specific effectors are shown in with yellow background. Effectors associated with the intracellular bacteria are shown underlined. **Table S4.** Frequency table for the effectors. The number represents in how many unique species and genera the homologues of an effector have been predicted to be present. Blank space against any effector implies its presence in only one bacterium. The numbers in the parenthesis indicates the number of bacteria in the background dataset which contain homologues of a given effector. Click here for file
