# Supplementary material for: New players in the same old game: a system level in silico study to predict type III secretion system and effector proteins in bacterial genomes reveals common themes in T3SS mediated pathogenesis
Source: BMC Res Notes. 2013 Jul 26;6:297. doi: 10.1186/1756-0500-6-297 (PMC3734048; doi:10.1186/1756-0500-6-297)
Supplement: Additional file 1 — Relative occurrences of T3SS proteins in bacterial kingdom. [file 1756-0500-6-297-S1.docx]

| E value | YscC | YscF | YscJ | YscN | YscQ | YscR | YscS | YscT | YscU | YscV |
| --- | --- | --- | --- | --- | --- | --- | --- | --- | --- | --- |
| 0.1 | 43.98 | 2.08 | 22.79 | 98.67 | 24.80 | 50.93 | 26.61 | 20.40 | 51.07 | 50.79 |
| 0.01 | 43.71 | 1.52 | 17.58 | 98.67 | 12.36 | 50.93 | 19.80 | 18.69 | 51.07 | 50.79 |
| 0.001 | 43.08 | 1.32 | 15.42 | 98.67 | 6.80 | 50.93 | 12.16 | 16.19 | 51.07 | 50.79 |
| 0.0001 | 42.11 | 1.32 | 13.82 | 98.67 | 3.75 | 50.93 | 7.30 | 14.10 | 51.07 | 50.79 |
| 0.00001 | 42.11 | 1.32 | 13.82 | 98.67 | 3.75 | 50.93 | 4.50 | 14.10 | 51.07 | 50.79 |

Table 1: Relative occurrences of T3SS proteins in bacterial kingdom.

The numbers represent the percentage of bacteria which are likely to contain a particular T3SS protein at a given ‘e’ value. T3SS proteins have been classified in four groups according to their relative occurrence in bacterial genome at different ‘e’ values. The first group, highlighted in the shade of cyan, consists of YscC, YscR, YscU and YscV is present in almost 50 % of the bacteria and are present at all the ‘e’. YscN, highlighted in red, is an ATPase. YscJ, YscS and YscT constitute the third group, highlighted in dark green. YscQ and YscF, highlighted in magenta represent the fourth group.
