# Supplementary material for: New players in the same old game: a system level in silico study to predict type III secretion system and effector proteins in bacterial genomes reveals common themes in T3SS mediated pathogenesis
Source: BMC Res Notes. 2013 Jul 26;6:297. doi: 10.1186/1756-0500-6-297 (PMC3734048; doi:10.1186/1756-0500-6-297)
Supplement: Additional file 2: Figure S1 — Phylogram of the 85 unique T3SS positive species based on the multiple sequence alignment of YscN, the most conserved T3SS component. Table S1. List of T3SS positive bacteria predicted in this study. It includes 152 unique serovars, 85 unique genera and 43 unique species. The newly identified bacteria are shown in gray shade. Table S2. Biochemical and physiological functions of the effectors. Four major classes are shown in different background colours. Effectors, which regulate the Rho GTPase, its downstream molecules or actin polymerization/de-polirization are shown with yellow background. Effectors with Cysteine protease activity are shown with cyan background. Effectors which modulate host cell function by ubiqutination of host factors are highlighted in magenta. Phosphatases are shown in dark green background. ‘NA’ implies the non-availability of data for a given effector. Table S3. Effector matrix for 152 T3SS positive bacteria. ‘1’ implies presence of a particular effector in a given bacterium and ‘0’ implies its absence. The plant specific effectors are shown in green background, whereas the animal specific effectors are shown in with yellow background. Effectors associated with the intracellular bacteria are shown underlined. Table S4. Frequency table for the effectors. The number represents in how many unique species and genera the homologues of an effector have been predicted to be present. Blank space against any effector implies its presence in only one bacterium. The numbers in the parenthesis indicates the number of bacteria in the background dataset which contain homologues of a given effector. [file 1756-0500-6-297-S2.pdf]

Legends to supplementary figures and tables:

Supplementary figure 1. Phylogram of the 85 unique T3SS positive species based on the multiple sequence alignment of YscN, the most conserved T3SS component.

Supplementary table 1. List of T3SS positive bacteria predicted in this study. It includes 152 unique serovars, 85 unique genera and 43 unique species. The newly identified bacteria are shown in gray shade.

Supplementary table 2. Biochemical and physiological functions of the effectors. Four major classes are shown in different background colours. Effectors, which regulate the Rho GTPase, its downstream molecules or actin polymerization/de-polirization are shown with yellow background. Effectors with Cysteine protease activity are shown with cyan background. Effectors which modulate host cell function by ubiqutination of host factors are highlighted in magenta. Phosphatases are shown in dark green background. 'NA' implies the non-availability of data for a given effector.

Supplementary table 3. Effector matrix for 152 T3SS positive bacteria. '1' implies presence of a particular effector in a given bacterium and '0' implies its absence. The plant specific effectors are shown in green background, whereas the animal specific effectors are shown in with yellow background. Effectors associated with the intracellular bacteria are shown underlined.

Supplementary table 4. Frequency table for the effectors. The number represents in how many unique species and genera the homologues of an effector have been predicted to be present. Blank space against any effector implies its presence in only one bacterium. The numbers in the parenthesis indicates the number of bacteria in the background dataset which contain homologues of a given effector.

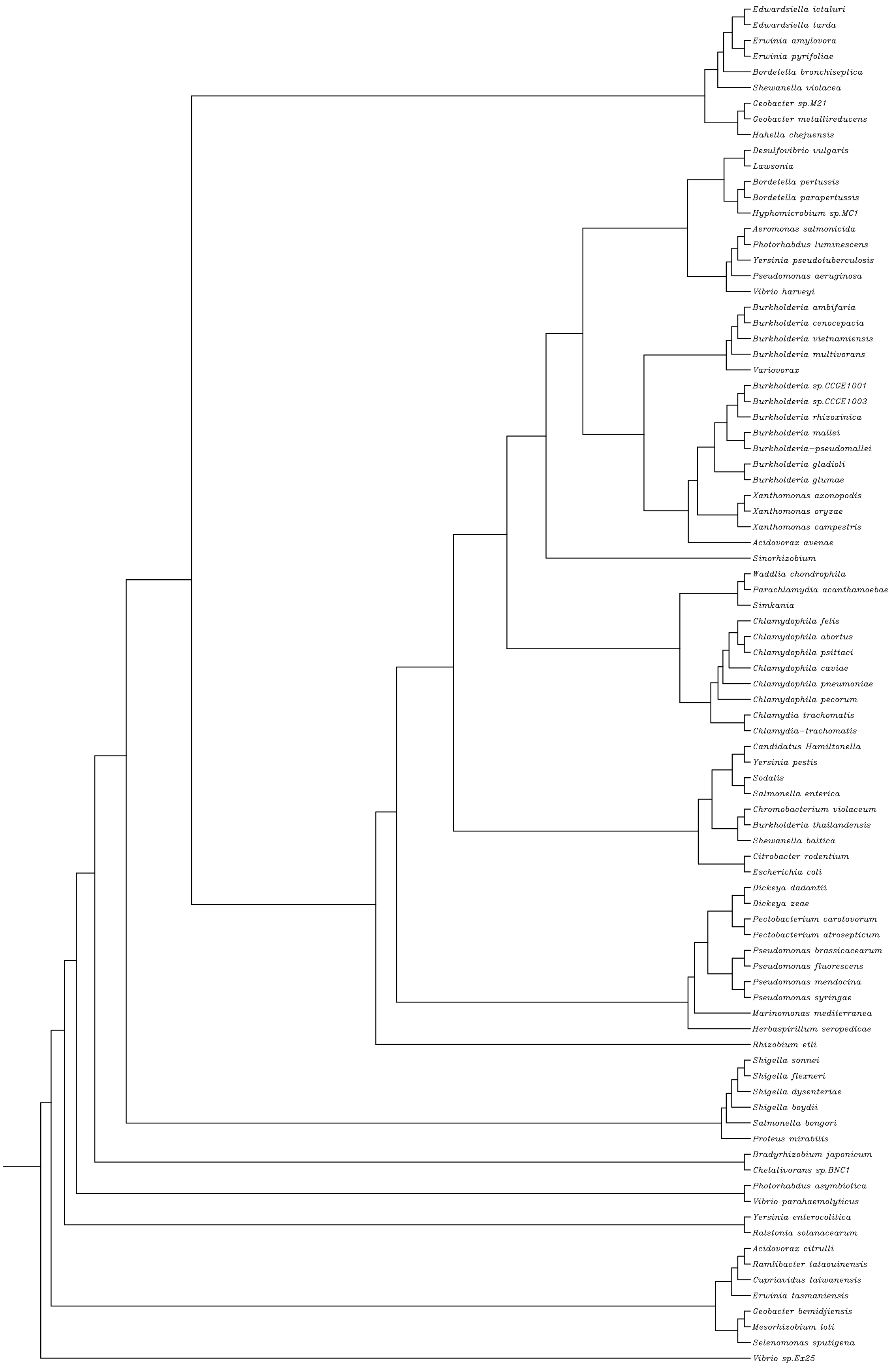
Edwardsiella ictaluri
Edwardsiella tarda
Erwinia amylovora
Erwinia pyrifoliae
Bordetella bronchiseptica
Shewanella violacea
Geobacter sp.M21
Geobacter metallireducens
Hahella chejuensis
Desulfovibrio vulgaris
Lawsonia
Bordetella pertussis
Bordetella parapertussis
Hyphomicrobium sp.MC1
Aeromonas salmonicida
Photorhabdus luminescens
Yersinia pseudotuberculosis
Pseudomonas aeruginosa
Vibrio harveyi
Burkholderia ambifaria
Burkholderia cenocepacia
Burkholderia vietnamiensis
Burkholderia multivorans
Variovorax
Burkholderia sp.CCGE1001
Burkholderia sp.CCGE1003
Burkholderia rhizoxinica
Burkholderia mallei
Burkholderia–pseudomallei
Burkholderia gladioli
Burkholderia glumae
Xanthomonas axonopodis
Xanthomonas oryzae
Xanthomonas campestris
Acidovorax avenae
Sinorhizobium
Waddlia chondrophila
Parachlamydia acanthamoebae
Simkania
Chlamydophila felis
Chlamydophila abortus
Chlamydophila psittaci
Chlamydophila caviae
Chlamydophila pneumoniae
Chlamydophila pecorum
Chlamydia trachomatis
Chlamydia–trachomatis
Candidatus Hamiltonella
Yersinia pestis
Sodalis
Salmonella enterica
Chromobacterium violaceum
Burkholderia thailandensis
Shewanella baltica
Citrobacter rodentium
Escherichia coli
Dickeya dadantii
Dickeya zeae
Pectobacterium carotovorum
Pectobacterium atrosepticum
Pseudomonas brassicacearum
Pseudomonas fluorescens
Pseudomonas mendocina
Pseudomonas syringae
Marinomonas mediterranea
Herbaspirillum seropedicae
Rhizobium etli
Shigella sonnei
Shigella flexneri
Shigella dysenteriae
Shigella boydii
Salmonella bongori
Proteus mirabilis
Bradyrhizobium japonicum
Chelativorans sp.BNC1
Photorhabdus asymbiotica
Vibrio parahaemolyticus
Yersinia enterocolitica
Ralstonia solanacearum
Acidovorax citrulli
Ramlibacter tataouinensis
Cupriavidus taiwanensis
Erwinia tasmaniensis
Geobacter bemidjiensis
Mesorhizobium loti
Selenomonas sputigena
Vibrio sp.Ex25

Supplementary table 1

Serovar	Species	Genera
Acidovorax avenae ATCC 19860	Acidovorax avenae	Acidovorax
Acidovorax citrulli AAC00 1	Acidovorax citrulli	Aeromonas
Aeromonas salmonicida A449	Aeromonas salmonicida	Bordetella
Bordetella bronchiseptica RB50	Bordetella bronchiseptica	Bradyrhizobium
Bordetella parapertussis 12822	Bordetella parapertussis	Burkholderia
Bordetella pertussis Tohama I	Bordetella pertussis	Candidatus
Bradyrhizobium japonicum USDA 110	Bradyrhizobium japonicum	Chelativorans
Burkholderia ambifaria AMMD	Burkholderia ambifaria	Chlamydophila
Burkholderia ambifaria MC40 6	Burkholderia CCGE1001	Chromobacterium
Burkholderia cenocepacia AU 1054	Burkholderia CCGE1003	Citrobacter
Burkholderia cenocepacia HI2424	Burkholderia cenocepacia	Cupriavidus
Burkholderia cenocepacia J2315	Burkholderia gladioli	Desulfovibrio
Burkholderia cenocepacia MC0 3	Burkholderia glumae	Dickeya
Burkholderia gladioli BSR3	Burkholderia mallei	Edwardsiella
Burkholderia glumae BGR1	Burkholderia multivorans	Erwinia
Burkholderia mallei ATCC 23344	Burkholderia pseudomallei	Escherichia
Burkholderia mallei NCTC 10229	Burkholderia rhizoxinica	Geobacter
Burkholderia mallei NCTC 10247	Burkholderia thailandensis	Hahella
Burkholderia multivorans ATCC 17616	Burkholderia vietnamiensis	Herbaspirillum
Burkholderia pseudomallei 1106a	Candidatus Hamiltonella	Hyphomicrobium
Burkholderia pseudomallei 1710b	Chelativorans BNC1/	Lawsonia
Burkholderia pseudomallei 668	Chlamydophila abortus	Marinomonas
Burkholderia pseudomallei K96243	Chlamydophila caviae	Mesorhizobium
Burkholderia rhizoxinica HKI 454	Chlamydophila felis	Parachlamydia
Burkholderia CCGE1001	Chlamydophila pecorum	Pectobacterium
Burkholderia CCGE1003	Chlamydophila pneumoniae	Photorhabdus
Burkholderia thailandensis E264	Chlamydophila psittaci	Proteus
Burkholderia vietnamiensis G4	Chromobacterium violaceum	Pseudomonas
Candidatus Hamiltonella defensa 5AT Acyrthosiphon pisum	Citrobacter rodentium	Ralstonia
Chelativorans BNC1/	Cupriavidus taiwanensis	Ramlibacter
Chlamydophila abortus S26 3	Desulfovibrio vulgaris	Rhizobium
Chlamydophila caviae GPIC	Dickeya dadantii	Salmonella
Chlamydophila felis Fe C 56	Dickeya zeae	Selenomonas
Chlamydophila pecorum E58	Edwardsiella ictaluri	Shewanella
Chlamydophila pneumoniae AR39	Edwardsiella tarda	Shigella
Chlamydophila pneumoniae CWL029	Erwinia amylovora	Simkania
Chlamydophila pneumoniae J138	Erwinia pyrifoliae	Sinorhizobium
Chlamydophila pneumoniae TW 183	Erwinia tasmaniensis	Sodalis
Chlamydophila psittaci 6BC	Escherichia coli	Variovorax
Chromobacterium violaceum ATCC 12472	Geobacter bemidjiensis	Vibrio
Citrobacter rodentium ICC168	Geobacter M21	Waddlia
Cupriavidus taiwanensis LMG 19424	Geobacter metallireducens	Xanthomonas
Desulfovibrio vulgaris Hildenborough	Hahella chejuensis	Yersinia
Desulfovibrio vulgaris Miyazaki F	Herbaspirillum seropedicae	
Desulfovibrio vulgaris DP4	Hyphomicrobium MC1	
Dickeya dadantii 3937	Lawsonia intracellularis	
Dickeya dadantii Ech586	Marinomonas mediterranea	
Dickeya zeae Ech1591	Mesorhizobium loti	
Edwardsiella ictaluri 93 146	Parachlamydia acanthamoebae	

Edwardsiella tarda EIB202	Pectobacterium atrosepticum	
Erwinia amylovora ATCC 49946	Pectobacterium carotovorum	
Erwinia amylovora CFBP1430	Photorhabdus asymbiotica	
Erwinia pyrifoliae Ep1 96	Photorhabdus luminescens	
Erwinia tasmaniensis Et1 99	Proteus mirabilis	
Escherichia coli 55989	Pseudomonas aeruginosa	
Escherichia coli E24377A	Pseudomonas brassicacearum	
Escherichia coli IAI1	Pseudomonas fluorescens	
Escherichia coli O103 H2 12009	Pseudomonas mendocina	
Escherichia coli O111 H 11128	Pseudomonas syringae	
Escherichia coli O127 H6 E2348 69	Ralstonia solanacearum	
Escherichia coli O157 H7 EC4115	Ramlibacter tataouinensis	
Escherichia coli O157 H7 EDL933	Rhizobium etli	
Escherichia coli O157 H7 Sakai	Salmonella bongori	
Escherichia coli O157 H7 TW14359	Salmonella enterica	
Escherichia coli O26 H11 11368	Selenomonas sputigena	
Escherichia coli O55 H7 CB9615	Shewanella baltica	
Escherichia coli SE11	Shewanella violacea	
Escherichia coli UMN026	Shigella boydii	
Geobacter bemidjiensis Bem	Shigella dysenteriae	
Geobacter metallireducens GS 15	Shigella flexneri	
Geobacter M21	Shigella sonnei	
Hahella chejuensis KCTC 2396	Simkania negevensis	
Herbaspirillum seropedicae SmR1	Sinorhizobium fredii	
Hyphomicrobium MC1	Sodalis glossinidius	
Lawsonia intracellularis PHE MN1 00	Variovorax paradoxus	
Marinomonas mediterranea MMB 1	Vibrio Ex25	
Mesorhizobium loti MAFF303099	Vibrio harveyi	
Parachlamydia acanthamoebae UV7	Vibrio parahaemolyticus	
Pectobacterium atrosepticum SCRI1043	Waddlia chondrophila	
Pectobacterium carotovorum PC1	Xanthomonas axonopodis	
Photorhabdus asymbiotica ATCC 43949	Xanthomonas campestris	
Photorhabdus luminescens laumondii TTO1	Xanthomonas oryzae	
Proteus mirabilis HI4320	Yersinia enterocolitica	
Pseudomonas aeruginosa LESB58	Yersinia pestis	
Pseudomonas aeruginosa PAO1	Yersinia pseudotuberculosis	
Pseudomonas aeruginosa UCBPP PA14		
Pseudomonas brassicacearum NFM421		
Pseudomonas fluorescens SBW25		
Pseudomonas mendocina ymp		
Pseudomonas syringae phaseolicola 1448A		
Pseudomonas syringae B728a		
Pseudomonas syringae tomato DC3000		
Ralstonia solanacearum GMI1000		
Ralstonia solanacearum MolK2		
Ralstonia solanacearum PSI07		
Ramlibacter tataouinensis TTB310		
Rhizobium etli CFN 42		
Salmonella bongori NCTC 12419		
Salmonella enterica arizonae serovar 62 z4 z23 RSK2980		
Salmonella enterica serovar Agona SL483		
Salmonella enterica serovar Choleraesuis SC B67		
Salmonella enterica serovar Dublin CT 02021853		
Salmonella enterica serovar Enteritidis P125109		
Salmonella enterica serovar Gallinarum 287 91		

Salmonella enterica serovar Heidelberg SL476		
Salmonella enterica serovar Newport SL254		
Salmonella enterica serovar Paratyphi A AKU 12601		
Salmonella enterica serovar Paratyphi A ATCC 9150		
Salmonella enterica serovar Paratyphi B SPB7		
Salmonella enterica serovar Paratyphi C RKS4594		
Salmonella enterica serovar Schwarzengrund CVM19633		
Salmonella enterica serovar Typhimurium LT2		
Salmonella enterica serovar Typhi CT18		
Salmonella enterica serovar Typhi Ty2		
Selenomonas sputigena ATCC 35185		
Shewanella baltica OS155		
Shewanella baltica OS195		
Shewanella violacea DSS12		
Shigella boydii CDC 3083 94		
Shigella dysenteriae Sd197		
Shigella flexneri 2a 301		
Shigella sonnei Ss046		
Simkania negevensis Z		
Sinorhizobium fredii NGR234		
Sodalis glossinidius morsitans		
Variovorax paradoxus EPS		
Vibrio harveyi ATCC BAA 1116		
Vibrio parahaemolyticus RIMD 2210633		
Vibrio Ex25		
Waddlia chondrophila WSU 86 1044		
Xanthomonas axonopodis citri 306		
Xanthomonas campestris 8004		
Xanthomonas campestris ATCC 33913		
Xanthomonas campestris B100		
Xanthomonas campestris vesicatoria 85 10		
Xanthomonas oryzae KACC10331		
Xanthomonas oryzae MAFF 311018		
Xanthomonas oryzae PXO99A		
Yersinia enterocolitica 8081		
Yersinia enterocolitica palearctica 105 5R r		
Yersinia pestis Angola		
Yersinia pestis Antiqua		
Yersinia pestis biovar Microtus 91001		
Yersinia pestis CO92		
Yersinia pestis Nepal516		
Yersinia pestis Pestoides F		
Yersinia pestis Z176003		
Yersinia pseudotuberculosis IP 31758		
Yersinia pseudotuberculosis IP 32953		
Yersinia pseudotuberculosis PB1		
Yersinia pseudotuberculosis YPIII		

Supplementary table 2

Name	Biochemical activity	Biological function
AexT.txt	RhoGAP	Cytoskeletal remodelling.
AvrA.txt	Cysteine protease	Modulate the activity of JNK pathway to inhibit apoptosis.
AvrBsT.txt	Cysteine protease	Remove sumo modification from defence related proteins in plant by protease activity.
AvrPphB.txt	Cysteine protease	Remove sumo modification from defence related proteins in plant by protease activity.
AvrPtoB.txt	Ub ligase	Suppress plant resistance.
BB4228.txt	NA	NA
BopA.txt	NA	Helps in escaping from phagosome which follows replication.
BopE.txt	Rho GEF	Cytoskeletal remodelling.
BPSS1516.txt	NA	NA
BPSS1539.txt	NA	NA
Cp0034.txt	NA	NA
EspF.txt	Binds to SNX9 via SH3 binding motif.	Membrane remodelling by recruitment of SNX9 onto PM.
EspG.txt	Increase active population of ARF GTPase, activation of P21 activated kinase activity.	Disruption of tight junction, inhibition of protein secretion via Golgi.
EspH.txt	Binds to RhoGEF and deactivate Rho	Cytoskeletal remodelling/focal adhesion disassembly, triggers cell detachment, activates caspase-3, and induces cytotoxicity.
EspZ.txt	Localizes to Mitochondrea and interacts with translocase in the inner membrane	Survival signal in host to defer the host cell death.
ExoS.txt	RhoGAP/ADP ribosylation	Cytoskeletal remodelling/inhibits phagocytosis.
ExoT.txt	RhoGAP/ADP ribosylation	Cytoskeletal remodelling.
ExoU.txt	Phospholipase	Cytotoxic/kills epithelial and other cell types.
ExoY.txt	Adenylate cyclase	Disrupt Cytoskeletal arrangement/inhibit phagocytosis of the bacteria into host cells.
GALA.txt	F box containing protein whcih recruits target proteins to ubiquitin conjugating proteins	F box is important for virulence.
HopptoD2.txt	Tyr Phosphatase	Suppress plant resistance.
HopptoN.txt	Cysterine protease	Suppress plant resistance.
Ibe.txt	Binds to IQGAP	Modulate Actin cytoskeleton and stabilizes PM pedestal for attachment of the bacteria to cell surface.
IcsB.txt	Compete with ATG5 for VirA binding with	Modulate Autophagy.
IncA.txt	Intracellular development	Inclusion body associated protein.

	of Chlamydia	
IncB.txt	NA	Inclusion membrane associated protein.
IncD.txt	NA	NA
IncE.txt	NA	NA
IncF.txt	NA	NA
IncG.txt	NA	NA
IpaA.txt	Actin depolymerization/binds to vinculin	Induce membrane ruffle by recycling Actin from stress fibre/Cytoskeletal remodelling.
IpaB.txt	Activate Caspase 1	Fragments golgi and affect cell surface receptor recycling.
IpaC.txt	Actin polymerization by activating Src kinase and Arp2/3	Invasion of Shigella into epithelial cells /Cytoskeletal remodelling.
IpaH.txt	Ub ligase/ubiquitination of MAP kinas	Block innate immune response possibly by down regulating MAPKK and downstream expression involved in innate response.
Ipgb.txt	Activate RhoG/RhoA	Induce membrane ruffles during bacterial invasion/Cytoskeletal remodelling.
IpgD.txt	Phosphatase/ Promotes formation of PI5P from PI(4,5)P2.	Causes membrane blebbing.
LopT.txt	Cysteine protease/	Inhibit phagocytosis by releasing RhoA Rac from PM by protease activity/Cytoskeletal remodelling.
LscZ.txt	NA	NA
MAP.txt	Activate CDC42	Induces transient filopodium formation at bacterial attachment sites during the early stages of EPEC infection/Cytoskeletal remodelling.
NleB.txt	Inhibits translocation of P65 component of NF-κβ in TNF-a mediated activation	Inhibits NF-κβ activation.
Orf31.txt	Activate Rho/RAC/CDC42	Extected to modulate Cytoskeletal remodelling.
OspC3.txt	NA	Post invasion virulence / helps in disruption of cell barrier and increase PMN migration through epithelial cells thereby increased inflammation.
OspF.txt	Phospho Thereonine lyase, inactivate MAP kinase/ERK/JNK	Block innate immune response by down regulating transcription of the subset of NF-κβ regulated genes.
OspG.txt	Ser/Thr Kinase/ Ubiquitination of Iκβα and degradation via proteosome	Block innate immune response /repression of NF-κβ.
PipB.txt	Targeted to late endosomes	Inflammatory response / Most likely maturation of SCB/SIF.
PopP1.txt	Cysteine protease	Most likely to regulate plant defence proteins. Homologue, Popp2 binds to a transcription

		factor which regulate genes in a defence pathway.
SifA.txt	Activate Rho/RAC/CDC42	Induce long Actin filaments, by getting (Fusion of endosomal compartment with the salmonella vacculoe) membrane from the endosomal membrane.
SipA.txt	Actin polymerization thus slow down Actin dynamics	Entry of Salmonella by inducing growth of filopodia and membrane ruffle which engulf the bacteria/Cytoskeletal rearrangement.
SipB.txt	Caspase 1 binding	Induce apoptosis into macrophage.
SipC.txt	Nucleate Actin polymer formation and bundle in vitro	Maturation of Salmonella vaccule.
SlrP.txt	Binds to DNAJ(chaperone and interferes with binding to its substrate. It also ubiquitinate .	Cause accumulation of unfolded proteins in the host and alter cellular redox potential
SopA.txt	Ubiquitination	Induce inflammation.
SopB.txt	Phosphatse/membrane modification	Activate Cdc42/Rac/entry into host cells/also have role in phagosome maturation/Cytoskeletal remodelling.
SopD.txt	Targetted to late endosomes	Crucial for invasion, membrane fission, macro-pinosome formation
SopE.txt	Rho GEF	Remodelling Cytoskeletal structure to enter host cell./ activate CDC42/Rac/entry into host cells.
SpiC.txt	NA	Inhibits the fusion of the SCV with Lysosome. It activates MAP kinase pathway in macrophage.
SptP.txt	Rho GAP	Seal the leak/ rebuild the Cytoskeleton as it was in the uninfected state/opposing activity of SopE/SopB.
SseF.txt	Salmonella maturation	Involved in Salmonella induced filaments/bacteria proliferation.
SseG.txt	Salmonella maturation	Involved in Salmonella induced filaments/bacterial proliferation.
SseJ.txt	Lipase	SCV biogenesis.
SseL.txt	Cysteine protease/Deubiquitination using Cysteine protease	Binds to the oxysterol binding protein and recruits to SCV which plays crucial role in Salmonella replication.
SspH1.txt	Ub ligase	Inhibits NF-κβ dependent gene expression.
TarP.txt	Polymerize Actin in concert with Arp2/3 protein complex	Internalization Chlamydia/Cytoskeletal remodelling.
TccP2.txt	Activate Wasp mediated Actin polymerization	Cytoskeletal remodelling /might play a role in attachment to the host cell.
Tir.txt	Recruits ShIP2 to promotes phosphatise.	Attachment of EPEC to host cell surface. by inducing Actin supported membrane protrusion, lamelipodia /Inhibits NF-κβ activation.
VirA.txt	Cysteine protease/	Microtubule degradation/Cytoskeletal

		remodelling.
VopA.txt	Cysteine protease/acetyl transferase	Acetylates a conserved lysine found in the catalytic loop of all kinases and blocks the binding of ATP, but not ADP, on the MKKs, resulting in an inactive phosphorylated kinase.
VopF.txt	Inhibit Actin polymerization	Disrupt tight cell junction by de-polymerizing cortical Actin and miss-localizing Zo-1/ protects against apoptosis.
VopQ.txt	Autophagy	Inhibits phagocytosis during infection.
VopS.txt	Deactivate Rho and Rac family of GTPases by Ampylating their effectors	Cell shape is lost, rounded up/Cytoskeletal remodelling.
VP1680.txt	NA	Activates MAP kinase pathway which in turn induces inflammation.
VP1683.txt	NA	NA
VP1686.txt	Activate RhoB	Actin remodelling to increase uptake of the bacteria by macrophage.
VP1698.txt	NA	NA
VPA0450.txt	Phosphatase	Membrane blebbing.
XopD.txt	Cysteine protease	XopD specifically interacts with MYB30, resulting in inhibition of the transcriptional activation of MYB30 VLCFA-related target genes and suppression of Arabidopsis defence.
YopE.txt	Rho GAP	Deactivate Rho GTPase /helps the host cell to revert back to the normal steady state for the Cytoskeletal structure /negatively regulate effector entry into the host .
YopH.txt	Tyrosine Phosphatase	Inhibits phagocytosis by macrophage by inactivating several host proteins which initiate phagocytosis/also inhibits integrin signalling/Cytoskeletal remodelling.
YopJ.txt	Cysteine protease/Inhibit MAPk and NF-κβ pathway	Anti inflammation activity.
YopK.txt	NA	Regulate effector entry into host cells from within the host cell/promotes virulence by preventing inflammasome recognition of the Type III secretion system.
YopO.txt	Ser/thr Kinase and GDI like domain	Inhibits Fcγ receptor mediated phagocytosis sequestering Rac1/Cytoskeletal remodelling.
YopT.txt	Cysteine protease/regulate Rho activity by cleaving the membrane bound Rho GTPase	Inhibit phagocytosis/Cytoskeletal remodelling.

Supplementary table 3

Effector	AexT	AvrA	AvrBsT	AvrPphB	AvrPtoB	BB4228	BPSS1516	BPSS1539	BopA	BopE	EspB	EspD	EspF	EspG	EspH	EspZ	ExoS	ExoT	ExoU	ExoY	GALA
Pseudomonas syringae	0	0	0	1	1	0	0	0	0	0	0	0	0	0	0	0	0	0	0	0	0
Acidovorax avenae	0	1	1	1	0	0	0	0	0	0	0	0	0	0	0	0	0	0	0	0	1
Acidovorax citrulli	0	1	1	1	0	0	0	0	0	0	0	0	0	0	0	0	0	0	0	0	1
Erwinia amylovora	0	0	1	0	0	0	0	0	0	0	0	0	0	0	0	0	0	0	0	0	0
Erwinia pyrifoliae	0	0	1	0	0	0	0	0	0	0	0	0	0	0	0	0	0	0	0	0	0
Ralstonia solanacearum	0	0	1	0	0	0	0	0	0	0	0	0	0	0	0	0	0	0	0	0	1
Sinorhizobium fredii	0	0	1	1	0	0	0	0	0	0	0	0	0	0	0	0	0	0	0	0	0
Xanthomonas campestris	0	1	1	0	0	0	0	0	0	0	0	0	0	0	0	0	0	0	0	0	0
Bradyrhizobium japonicum	0	0	0	1	0	0	0	0	0	0	0	0	0	0	0	0	0	0	0	0	0
Dickeya dadantii	0	0	0	0	0	0	0	0	0	0	0	0	0	0	0	0	0	0	0	0	0
Dickeya zeae	0	0	0	0	0	0	0	0	0	0	0	0	0	0	0	0	0	0	0	0	0
Erwinia tasmaniensis	0	0	0	0	0	0	0	0	0	0	0	0	0	0	0	0	0	0	0	0	0
Herbaspirillum seropedicae	0	0	0	0	0	0	0	0	0	0	0	0	0	0	0	0	0	0	0	0	0
Mesorhizobium loti	0	0	0	0	0	0	0	0	0	0	0	0	0	0	0	0	0	0	0	0	0
Pectobacterium atrosepticum	0	0	0	0	0	0	0	0	0	0	0	0	0	0	0	0	0	0	0	0	0
Pectobacterium carotovorum	0	0	0	0	0	0	0	0	0	0	0	0	0	0	0	0	0	0	0	0	0
Pseudomonas brassicacearum	0	0	0	0	0	0	0	0	0	0	0	0	0	0	0	0	0	0	0	0	0
Pseudomonas fluorescens	0	0	0	0	0	0	0	0	0	0	0	0	0	0	0	0	0	0	1	1	0
Rhizobium etli	0	0	0	0	0	0	0	0	0	0	0	0	0	0	0	0	0	0	0	0	0
Xanthomonas axonopodis	0	0	0	0	0	0	0	0	0	0	0	0	0	0	0	0	0	0	0	0	0
Xanthomonas oryzae	0	0	0	0	0	0	0	0	0	0	0	0	0	0	0	0	0	0	0	0	0
Salmonella bongori	0	1	1	0	0	0	0	0	0	1	0	0	0	0	0	0	0	1	0	0	0
Salmonella enterica	0	1	1	0	0	0	0	0	0	1	0	0	0	0	0	0	0	0	0	0	0
Yersinia enterocolitica	0	1	1	0	0	0	0	0	0	0	0	0	0	0	0	0	1	1	0	0	0
Yersinia pestis	0	1	1	0	0	0	0	0	0	0	0	0	0	0	0	0	0	1	0	1	0
Yersinia pseudotuberculosis	0	1	1	0	0	0	0	0	0	0	0	0	0	0	0	0	0	1	0	1	0
Burkholderia ambifaria	0	0	0	0	0	0	0	0	0	0	0	0	0	0	0	0	0	0	0	0	0
Burkholderia CCGE1001	0	0	0	0	0	0	0	0	0	0	0	0	0	0	0	0	0	0	0	0	0

Burkholderia CCGE1003	0	0	0	0	0	0	0	0	0	0	0	0	0	0	0	0	0	0	0	0	0
Burkholderia cenocepacia	0	0	0	0	0	0	0	0	0	0	0	0	0	0	0	0	0	0	0	0	0
Burkholderia gladioli	0	0	0	0	0	0	0	0	0	0	0	0	0	0	0	0	0	0	0	0	1
Burkholderia glumae	0	0	0	0	0	0	0	0	0	0	0	0	0	0	0	0	0	0	0	0	0
Burkholderia mallei	0	0	0	0	0	0	1	1	1	1	0	0	0	0	0	0	0	0	0	0	0
Burkholderia multivorans	0	0	0	0	0	0	0	0	0	0	0	0	0	0	0	0	0	0	0	0	0
Burkholderia pseudomallei	0	0	0	0	0	0	1	1	1	1	0	0	0	0	0	0	0	0	0	0	0
Burkholderia rhizoxinica	0	0	0	0	0	0	0	0	0	0	0	0	0	0	0	0	0	0	0	0	1
Burkholderia thailandensis	0	0	0	0	0	0	0	1	1	1	0	0	0	0	0	0	0	0	0	0	0
Burkholderia vietnamiensis	0	0	0	0	0	0	0	0	0	0	0	0	0	0	0	0	0	0	0	0	0
Chlamydophila abortus	0	0	0	0	0	0	0	0	0	0	0	0	0	0	0	0	0	0	0	0	0
Chlamydophila caviae	0	0	0	0	0	0	0	0	0	0	0	0	0	0	0	0	0	0	0	0	0
Chlamydophila felis	0	0	0	0	0	0	0	0	0	0	0	0	0	0	0	0	0	0	0	0	0
Chlamydophila pecorum	0	0	0	0	0	0	0	0	0	0	0	0	0	0	0	0	0	0	0	0	0
Chlamydophila pneumoniae	0	0	0	0	0	0	0	0	0	0	0	0	0	0	0	0	0	0	0	0	0
Chlamydophila psittaci	0	0	0	0	0	0	0	0	0	0	0	0	0	0	0	0	0	0	0	0	0
Pseudomonas aeruginosa	1	0	0	0	0	0	0	0	0	0	0	0	0	0	0	0	1	1	1	1	0
Shigella boydii	0	0	0	0	0	0	0	0	1	0	0	0	0	1	0	0	0	0	0	0	0
Shigella dysenteriae	0	0	0	0	0	0	0	0	1	0	0	0	0	1	0	0	0	0	0	0	0
Shigella flexneri	0	0	0	0	0	0	0	0	1	0	0	0	0	1	0	0	0	0	0	0	0
Shigella sonnei	0	0	0	0	0	0	0	0	1	0	0	0	0	1	0	0	0	0	0	0	0
Photorhabdus luminescens	0	0	0	0	0	0	0	0	0	0	0	0	0	0	0	0	0	0	0	0	0
Edwardsiella ictaluri	0	0	0	0	0	0	0	0	0	0	0	0	0	0	0	0	0	0	0	1	0
Shewanella baltica	0	0	0	0	0	0	0	0	1	0	0	0	0	0	0	0	0	0	0	0	0
Waddlia chondrophila	0	0	0	0	0	0	0	0	0	0	0	0	0	0	0	0	0	0	0	0	0
Parachlamydia acanthamoebae	0	0	0	0	0	0	0	0	0	0	0	0	0	0	0	0	0	0	0	0	0
Escherichia coli	0	0	0	0	0	0	0	0	0	0	1	1	1	1	1	1	0	0	0	0	0
Marinomonas mediterranea	0	0	1	0	0	0	0	0	0	0	0	0	0	0	0	0	0	0	0	0	0
Vibrio parahaemolyticus	1	1	1	0	0	0	0	0	0	0	0	0	0	0	0	0	1	1	0	0	0
Aeromonas salmonicida	1	0	0	0	0	0	0	0	0	0	0	0	0	0	0	0	1	1	0	0	0
Bordetella bronchiseptica	0	0	0	0	0	1	0	0	0	0	0	0	0	0	0	0	0	0	0	1	0
Bordetella parapertussis	0	0	0	0	0	1	0	0	0	0	0	0	0	0	0	0	0	0	0	1	0

Bordetella pertussis	0	0	0	0	0	1	0	0	0	0	0	0	0	0	0	0	0	0	0	1	0
Candidatus Hamiltonella	1	0	0	0	0	0	0	0	0	0	0	0	0	0	0	0	1	1	0	1	0
Chromobacterium violaceum	0	0	0	0	0	0	0	0	0	1	0	0	0	0	0	0	0	0	0	0	0
Citrobacter rodentium	0	0	0	0	0	0	0	0	0	0	1	1	1	1	1	1	0	0	0	0	0
Cupriavidus taiwanensis	0	0	0	0	0	0	0	0	0	0	0	0	0	0	0	0	0	0	0	0	0
Desulfovibrio vulgaris	0	0	0	0	0	0	0	0	0	0	0	0	0	0	0	0	0	0	0	0	0
Edwardsiella tarda	0	0	0	0	0	0	0	0	0	0	0	0	0	0	0	0	0	0	0	0	0
Geobacter bemidjiensis	0	0	0	0	0	0	0	0	0	0	0	0	0	0	0	0	0	0	0	0	0
Geobacter M21	0	0	0	0	0	0	0	0	0	0	0	0	0	0	0	0	0	0	0	0	0
Geobacter metallireducens	0	0	0	0	0	0	0	0	0	0	0	0	0	0	0	0	0	0	0	0	0
Hahella chejuensis	0	0	0	0	0	0	0	0	0	0	0	0	0	0	0	0	0	0	0	0	0
Hyphomicrobium MC1	0	0	0	0	0	0	0	0	0	0	0	0	0	0	0	0	0	0	0	0	0
Lawsonia intracellularis	0	0	0	0	0	0	0	0	0	0	0	0	0	0	0	0	0	0	0	0	0
Photorhabdus asymbiotica	0	0	0	0	0	0	0	0	0	0	0	0	0	0	0	0	0	0	1	0	0
Proteus mirabilis	0	0	0	0	0	0	0	0	0	0	0	0	0	0	0	0	0	0	0	0	0
Pseudomonas mendocina	0	0	0	0	0	0	0	0	0	0	0	0	0	0	0	0	0	0	0	0	0
Ramlibacter tataouinensis	0	0	0	0	0	0	0	0	0	0	0	0	0	0	0	0	0	0	0	0	0
Selenomonas sputigena	0	0	0	0	0	0	0	0	0	0	0	0	0	0	0	0	0	0	0	0	0
Shewanella violacea	0	0	0	0	0	0	0	0	0	0	0	0	0	0	0	0	0	0	0	0	0
Simkania negevensis	0	0	0	0	0	0	1	0	0	0	0	0	0	0	0	0	0	0	0	0	0
Sodalis glossinidius	0	0	0	0	0	0	0	0	0	0	0	0	0	0	0	0	0	0	0	0	0
Variovorax paradoxus	0	0	0	0	0	0	0	0	0	0	0	0	0	0	0	0	0	0	0	0	0
Vibrio Ex25	0	0	0	0	0	0	0	0	0	0	0	0	0	0	0	0	0	0	0	0	0
Vibrio harveyi	0	0	0	0	0	0	0	0	0	0	0	0	0	0	0	0	0	0	0	0	0
**effector**	**OspG**	**PipB**	**PopP1**	**SifA**	**SipA**	**SipB**	**SipC**	**SlrP**	**SopA**	**SopB**	**SopD**	**SopE**	**SpiC**	**SptP**	**SseF**	**SseG**	**SseJ**	**SseL**	**SspH1**	**TarP**	**TccP2**
Pseudomonas syringae	0	0	0	0	0	0	0	0	0	0	0	0	0	0	0	0	0	0	1	0	0
Acidovorax avenae	0	0	1	0	0	0	0	0	0	0	0	0	0	0	0	0	0	0	0	0	0
Acidovorax citrulli	0	0	1	0	0	0	0	0	0	0	0	0	0	0	0	0	0	0	0	0	0
Erwinia amylovora	1	0	0	0	0	1	0	0	1	0	0	0	0	1	0	0	0	0	0	0	0
Erwinia pyrifoliae	0	0	0	0	0	0	0	0	1	0	0	0	0	1	0	0	0	0	0	0	0
Ralstonia solanacearum	0	0	1	0	0	0	0	0	0	0	0	0	0	0	0	0	0	0	1	0	0

Sinorhizobium fredii	0	1	1	0	0	0	0	1	0	0	0	0	0	0	0	0	0	0	1	0	0
Xanthomonas campestris	0	0	1	0	0	0	0	0	0	0	0	0	0	0	0	0	0	0	0	0	0
Bradyrhizobium japonicum	0	1	0	0	0	0	0	1	0	0	0	0	0	0	0	0	0	0	1	0	0
Dickeya dadantii	0	0	0	0	0	0	0	0	0	0	0	0	0	0	0	0	0	0	0	0	0
Dickeya zeae	0	1	0	0	0	0	0	0	0	0	0	0	0	0	0	0	0	0	0	0	0
Erwinia tasmaniensis	0	0	0	0	0	1	0	0	0	0	0	0	0	1	0	0	0	0	0	0	0
Herbaspirillum seropedicae	0	0	0	0	0	0	0	0	0	0	0	0	0	0	0	0	0	0	0	0	0
Mesorhizobium loti	0	1	0	0	0	0	0	0	1	0	0	0	0	0	0	0	0	0	0	0	0
Pectobacterium atrosepticum	0	0	0	0	0	0	0	0	0	0	0	0	0	0	0	0	0	0	0	0	0
Pectobacterium carotovorum	0	0	0	0	0	0	0	0	0	0	0	0	0	0	0	0	0	0	0	0	0
Pseudomonas brassicacearum	0	1	0	0	0	0	0	1	0	0	0	0	0	0	0	0	0	0	1	0	0
Pseudomonas fluorescens	0	1	0	0	0	0	0	1	0	0	0	0	0	0	0	0	0	0	1	0	0
Rhizobium etli	0	1	0	0	0	0	0	0	0	0	0	0	0	0	0	0	0	0	0	0	0
Xanthomonas axonopodis	0	0	0	0	0	0	0	0	0	0	0	0	0	0	0	0	0	0	0	0	0
Xanthomonas oryzae	0	0	0	0	0	0	0	0	0	0	0	0	0	0	0	0	0	0	0	0	0
Salmonella bongori	1	0	0	0	1	1	1	1	1	1	1	1	0	1	0	0	0	0	1	0	0
Salmonella enterica	0	1	0	1	1	1	1	1	1	1	1	1	1	1	1	1	1	1	1	0	0
Yersinia enterocolitica	1	0	1	0	0	0	0	1	1	0	0	0	0	1	0	0	1	0	1	0	0
Yersinia pestis	0	0	0	0	0	0	0	1	1	0	0	0	0	1	0	0	0	0	1	0	0
Yersinia pseudotuberculosis	1	1	0	0	0	0	0	1	1	0	0	0	0	1	0	0	0	0	1	0	0
Burkholderia ambifaria	0	0	0	0	0	0	0	0	0	0	0	0	0	0	0	0	0	0	0	0	0
Burkholderia CCGE1001	0	0	0	0	0	0	0	0	0	0	0	0	0	0	0	0	0	0	0	0	0
Burkholderia CCGE1003	0	0	0	0	0	0	0	0	0	0	0	0	0	0	0	0	0	0	0	0	0
Burkholderia cenocepacia	0	0	0	0	0	0	0	1	0	0	0	0	0	0	0	0	0	0	0	0	0
Burkholderia gladioli	0	0	0	0	0	0	0	0	0	0	0	0	0	0	0	0	0	0	0	0	0
Burkholderia glumae	0	1	0	0	0	0	0	0	0	0	0	0	0	0	0	0	0	0	0	0	0
Burkholderia mallei	0	1	0	0	0	1	0	0	0	0	0	1	0	0	0	0	0	0	0	0	0
Burkholderia multivorans	0	0	0	0	0	0	0	0	0	0	0	0	0	0	0	0	1	0	0	0	0
Burkholderia pseudomallei	0	1	0	0	0	1	0	0	0	0	0	1	0	0	0	0	0	0	0	0	0
Burkholderia rhizoxinica	0	0	0	0	0	0	0	0	0	0	0	0	0	0	0	0	1	0	0	0	0
Burkholderia thailandensis	0	1	0	0	0	1	0	0	0	0	0	1	0	0	0	0	0	0	0	0	0
Burkholderia vietnamiensis	0	0	0	0	0	0	0	0	0	0	0	0	0	0	0	0	0	0	0	0	0

Chlamydophila abortus	0	0	0	0	0	0	0	0	0	0	0	0	0	0	0	0	0	0	0	1	0
Chlamydophila caviae	0	0	0	0	0	0	0	0	0	0	0	0	0	0	0	0	0	0	0	1	0
Chlamydophila felis	0	0	0	0	0	0	0	0	0	0	0	0	0	0	0	0	0	0	0	1	0
Chlamydophila pecorum	0	0	0	0	0	0	0	0	0	0	0	0	0	0	0	0	0	0	0	1	0
Chlamydophila pneumoniae	0	0	0	0	0	0	0	0	0	0	0	0	0	0	0	0	0	0	0	1	0
Chlamydophila psittaci	0	0	0	0	0	0	0	0	0	0	0	0	0	0	0	0	0	0	0	1	0
Pseudomonas aeruginosa	0	1	0	0	0	0	0	1	0	0	0	0	0	0	0	0	0	0	1	0	0
Shigella boydii	1	0	0	0	1	1	1	1	1	1	0	0	0	0	0	0	0	1	1	0	0
Shigella dysenteriae	1	0	0	0	1	1	1	1	1	1	0	0	0	0	0	0	0	1	1	0	0
Shigella flexneri	1	1	0	0	1	1	1	1	1	1	0	0	0	0	0	0	0	0	1	0	0
Shigella sonnei	1	0	0	0	1	1	1	1	1	1	0	0	0	0	0	0	0	1	1	0	0
Photorhabdus luminescens	0	0	0	0	0	0	0	0	0	0	0	0	0	0	0	0	0	0	0	0	0
Edwardsiella ictaluri	0	1	0	1	0	0	0	1	0	0	1	0	0	0	0	0	1	0	1	0	0
Shewanella baltica	0	0	0	0	0	0	0	0	0	0	0	0	0	0	0	0	1	0	0	0	0
Waddlia chondrophila	0	0	0	0	0	0	0	0	0	0	0	0	0	0	0	0	0	0	0	0	0
Parachlamydia acanthamoebae	0	0	0	0	0	0	0	0	0	0	0	0	0	0	0	0	0	0	0	0	0
Escherichia coli	1	1	0	0	0	0	0	1	1	0	0	0	0	0	0	0	0	1	1	0	1
Marinomonas mediterranea	0	1	0	0	0	0	0	0	0	0	0	0	0	0	0	0	0	0	1	0	0
Vibrio parahaemolyticus	0	1	0	0	0	0	0	0	0	0	0	0	0	0	0	0	1	0	0	0	0
Aeromonas salmonicida	0	1	0	0	0	0	0	0	0	0	0	0	0	1	0	0	1	0	0	0	0
Bordetella bronchiseptica	0	1	0	0	0	0	0	0	0	0	0	0	0	0	0	0	0	0	0	0	0
Bordetella parapertussis	0	1	0	0	0	0	0	0	0	0	0	0	0	0	0	0	0	0	0	0	0
Bordetella pertussis	0	0	0	0	0	0	0	0	0	0	0	0	0	0	0	0	0	0	0	0	0
Candidatus Hamiltonella	0	1	0	0	0	1	0	0	0	0	0	0	0	1	0	0	0	0	0	0	0
Chromobacterium violaceum	0	0	0	0	1	1	1	0	0	0	1	1	0	1	0	0	1	0	0	0	0
Citrobacter rodentium	1	0	0	0	0	0	0	0	1	0	0	0	0	0	0	0	0	0	0	0	0
Cupriavidus taiwanensis	0	0	0	0	0	0	0	0	0	0	0	0	0	0	0	0	0	0	0	0	0
Desulfovibrio vulgaris	0	0	0	0	0	0	0	0	0	0	0	0	0	0	0	0	0	0	0	0	0
Edwardsiella tarda	0	0	0	0	0	0	0	0	0	0	0	0	0	0	0	0	0	0	0	0	0
Geobacter bemidjiensis	0	1	0	0	0	0	0	0	0	0	0	0	0	0	0	0	0	0	0	0	0
Geobacter M21	0	0	0	0	0	0	0	0	0	0	0	0	0	0	0	0	0	0	0	0	0
Geobacter metallireducens	0	1	0	0	0	0	0	0	0	0	0	0	0	0	0	0	0	0	0	0	0

Hahella chejuensis	0	0	0	0	0	0	0	0	0	0	0	0	0	0	0	0	0	0	1	0	0
Hyphomicrobium MC1	0	0	0	0	0	0	0	0	0	0	0	0	0	0	0	0	0	0	0	0	0
Lawsonia intracellularis	0	0	0	0	0	0	0	0	0	0	0	0	0	0	0	0	0	0	0	0	0
Photorhabdus asymbiotica	0	1	0	0	0	0	0	0	0	1	0	0	0	0	0	0	0	0	0	0	0
Proteus mirabilis	0	0	0	0	0	0	0	0	0	0	0	0	0	0	0	0	0	1	0	0	0
Pseudomonas mendocina	0	1	0	0	0	0	0	0	0	0	0	0	0	0	0	0	0	0	0	0	0
Ramlibacter tataouinensis	0	0	0	0	0	0	0	0	0	0	0	0	0	0	0	0	0	0	0	0	0
Selenomonas sputigena	0	0	0	0	0	0	0	0	0	0	0	0	0	0	0	0	0	0	0	0	0
Shewanella violacea	0	1	0	0	0	0	0	0	0	0	0	0	0	0	0	0	0	0	0	0	0
Simkania negevensis	0	1	0	0	0	0	0	0	0	0	0	0	0	0	0	0	0	0	0	0	0
Sodalis glossinidius	0	1	0	0	0	1	0	1	0	1	1	0	1	0	0	0	0	0	1	0	0
Variovorax paradoxus	0	1	0	0	0	0	0	0	0	0	0	0	0	0	0	0	0	0	0	0	0
Vibrio Ex25	0	0	0	0	0	0	0	0	0	0	0	0	0	0	0	0	0	0	0	0	0
Vibrio harveyi	0	0	0	0	0	0	0	1	0	0	0	0	0	0	0	0	1	0	0	0	0

**effector**	**HopptoD2**	**HopptoN**	**IcsB**	**IncA**	**IncB(4)**	**IncD**	**IncE**	**IncF**	**IncG**	**IpaA**	**IpaB**	**IpaC**	**IpaH**	**IpgD**	**Ipgb**	**LopT**	**LscZ**	**MAP**	**NleB**	**OspC**	**OspF**
Pseudomonas syringae	1	1	0	0	0	0	0	0	0	0	0	0	0	0	0	0	0	0	0	0	1
Acidovorax avenae	1	1	0	0	0	0	0	0	0	0	0	0	0	0	0	0	0	0	0	0	0
Acidovorax citrulli	1	1	0	0	0	0	0	0	0	0	0	0	0	0	0	0	0	0	0	0	0
Erwinia amylovora	0	0	0	0	0	0	0	0	0	0	1	0	0	0	0	0	0	0	0	0	0
Erwinia pyrifoliae	0	0	0	0	0	0	0	0	0	0	1	0	0	0	0	0	0	0	0	0	0
Ralstonia solanacearum	0	0	0	0	0	0	0	0	0	0	0	0	0	0	0	0	0	0	0	0	0
Sinorhizobium fredii	0	0	0	0	0	0	0	0	0	0	0	0	0	0	0	0	0	0	0	0	0
Xanthomonas campestris	1	1	0	0	0	0	0	0	0	0	0	0	0	0	0	0	0	0	0	0	0
Bradyrhizobium japonicum	0	0	0	0	0	0	0	0	0	0	0	0	0	0	0	0	0	0	0	0	0
Dickeya dadantii	0	0	0	0	0	0	0	0	0	0	0	0	0	0	0	0	0	0	0	0	0
Dickeya zeae	0	0	0	0	0	0	0	0	0	0	0	0	0	0	0	0	0	0	0	0	0
Erwinia tasmaniensis	0	0	0	0	0	0	0	0	0	0	1	0	0	0	0	0	0	0	0	0	0
Herbaspirillum seropedicae	0	0	0	0	0	0	0	0	0	0	0	0	0	0	0	0	0	0	0	0	0
Mesorhizobium loti	0	0	0	0	0	0	0	0	0	0	0	0	0	0	0	0	0	0	0	0	0
Pectobacterium atrosepticum	0	0	0	0	0	0	0	0	0	0	0	0	0	0	0	0	0	0	0	0	0
Pectobacterium carotovorum	0	0	0	0	0	0	0	0	0	0	0	0	0	0	0	0	0	0	0	0	0

Pseudomonas brassicacearum	0	0	0	0	0	0	0	0	0	0	0	0	0	0	0	0	0	0	0	0	0
Pseudomonas fluorescens	0	0	0	0	0	0	0	0	0	0	0	0	0	0	0	0	0	0	0	1	0
Rhizobium etli	0	0	0	0	0	0	0	0	0	0	0	0	0	0	0	0	0	0	0	0	0
Xanthomonas axonopodis	0	0	0	0	0	0	0	0	0	0	0	0	0	0	0	0	0	0	0	0	0
Xanthomonas oryzae	0	0	0	0	0	0	0	0	0	0	0	0	0	0	0	0	0	0	0	0	0
Salmonella bongori	0	0	0	0	0	0	0	0	0	1	1	1	0	1	0	0	0	0	0	1	0
Salmonella enterica	0	0	0	0	0	0	0	0	0	1	1	1	0	1	0	0	0	0	1	0	1
Yersinia enterocolitica	0	0	0	0	0	0	0	0	0	0	0	0	0	0	0	1	0	0	0	0	0
Yersinia pestis	0	0	0	0	0	0	0	0	0	0	0	0	0	0	0	1	0	0	0	0	0
Yersinia pseudotuberculosis	0	0	0	0	0	0	0	0	0	0	0	0	0	0	0	1	0	0	0	0	0
Burkholderia ambifaria	0	0	0	0	0	0	0	0	0	0	0	0	0	0	0	0	0	0	0	0	0
Burkholderia CCGE1001	0	0	0	0	0	0	0	0	0	0	0	0	0	0	0	0	0	0	0	0	0
Burkholderia CCGE1003	0	0	0	0	0	0	0	0	0	0	0	0	0	0	0	0	0	0	0	0	0
Burkholderia cenocepacia	0	0	0	0	0	0	0	0	0	0	0	0	0	0	0	0	0	0	0	0	0
Burkholderia gladioli	0	0	0	0	0	0	0	0	0	0	0	0	0	0	0	0	0	0	0	0	0
Burkholderia glumae	0	0	0	0	0	0	0	0	0	0	0	0	0	0	0	0	0	0	0	0	0
Burkholderia mallei	0	0	1	0	0	0	0	0	0	0	1	0	0	0	0	0	0	0	0	0	0
Burkholderia multivorans	0	0	0	0	0	0	0	0	0	0	0	0	0	0	0	0	0	0	0	0	0
Burkholderia pseudomallei	0	0	1	0	0	0	0	0	0	0	1	0	0	0	0	0	0	0	0	0	0
Burkholderia rhizoxinica	0	0	0	0	0	0	0	0	0	0	0	0	0	0	0	0	0	0	0	0	0
Burkholderia thailandensis	0	0	1	0	0	0	0	0	0	0	1	0	0	0	0	0	0	0	0	0	0
Burkholderia vietnamiensis	0	0	0	0	0	0	0	0	0	0	0	0	0	0	0	0	0	0	0	0	0
Chlamydophila abortus	0	0	0	0	1	0	0	0	0	0	0	0	0	0	0	0	0	0	0	0	0
Chlamydophila caviae	0	0	0	0	1	0	0	0	0	0	0	0	0	0	0	0	0	0	0	0	0
Chlamydophila felis	0	0	0	0	1	0	0	0	0	0	0	0	0	0	0	0	0	0	0	0	0
Chlamydophila pecorum	0	0	0	0	0	0	0	0	0	0	0	0	0	0	0	0	0	0	0	0	0
Chlamydophila pneumoniae	0	0	0	0	1	0	0	0	0	0	0	0	0	0	0	0	0	0	0	0	0
Chlamydophila psittaci	0	0	0	0	0	0	0	0	0	0	0	0	0	0	0	0	0	0	0	0	0
Pseudomonas aeruginosa	0	0	0	0	0	0	0	0	0	0	0	0	0	0	0	0	1	0	0	0	0
Shigella boydii	0	0	1	0	0	0	0	0	0	1	1	1	1	1	1	0	0	1	0	1	1
Shigella dysenteriae	0	0	1	0	0	0	0	0	0	1	1	1	1	1	1	0	0	1	0	1	1
Shigella flexneri	0	0	1	0	0	0	0	0	0	1	1	1	1	1	1	0	0	1	0	1	1

Shigella sonnei	0	0	1	0	0	0	0	0	0	1	1	1	1	1	1	0	0	1	0	1	1
Photorhabdus luminescens	0	0	0	0	0	0	0	0	0	0	0	0	0	0	0	1	1	0	0	0	0
Edwardsiella ictaluri	0	0	0	0	0	0	0	0	0	0	0	0	0	0	0	0	0	0	0	0	1
Shewanella baltica	0	0	0	0	0	0	0	0	0	0	0	0	0	0	0	0	0	0	0	0	0
Waddlia chondrophila	1	1	0	0	0	0	0	0	0	0	0	0	0	0	0	0	0	0	0	0	0
Parachlamydia acanthamoebae	1	1	0	0	0	0	0	0	0	0	0	0	0	0	0	0	0	0	0	0	0
Escherichia coli	0	0	0	0	0	0	0	0	0	0	0	0	1	0	1	0	0	1	1	0	0
Marinomonas mediterranea	0	0	0	0	0	0	0	0	0	0	0	0	0	0	0	0	0	0	0	0	0
Vibrio parahaemolyticus	0	0	0	0	0	0	0	0	0	0	0	0	0	0	0	0	0	0	0	1	0
Aeromonas salmonicida	0	0	0	0	0	0	0	0	0	0	0	0	0	0	0	0	1	0	0	0	0
Bordetella bronchiseptica	0	0	0	0	0	0	0	0	0	0	0	0	0	0	0	0	0	0	0	0	0
Bordetella parapertussis	0	0	0	0	0	0	0	0	0	0	0	0	0	0	0	0	0	0	0	0	0
Bordetella pertussis	0	0	0	0	0	0	0	0	0	0	0	0	0	0	0	0	0	0	0	0	0
Candidatus Hamiltonella	0	0	0	0	0	0	0	0	0	0	0	0	0	0	0	1	0	0	0	0	0
Chromobacterium violaceum	0	0	0	0	0	0	0	0	0	1	1	1	0	0	0	0	0	0	0	1	1
Citrobacter rodentium	0	0	0	0	0	0	0	0	0	0	0	0	0	0	1	0	0	1	1	0	0
Cupriavidus taiwanensis	0	0	0	0	0	0	0	0	0	0	0	0	0	0	0	0	0	0	0	0	0
Desulfovibrio vulgaris	0	0	0	0	0	0	0	0	0	0	0	0	0	0	0	0	0	0	0	0	0
Edwardsiella tarda	0	0	0	0	0	0	0	0	0	0	0	0	0	0	0	0	0	0	0	0	1
Geobacter bemidjiensis	0	0	0	0	0	0	0	0	0	0	0	0	0	0	0	0	0	0	0	0	0
Geobacter M21	0	0	0	0	0	0	0	0	0	0	0	0	0	0	0	0	0	0	0	0	0
Geobacter metallireducens	0	0	0	0	0	0	0	0	0	0	0	0	0	0	0	0	0	0	0	0	0
Hahella chejuensis	0	0	0	0	0	0	0	0	0	0	0	0	0	0	0	1	0	0	0	0	0
Hyphomicrobium MC1	0	0	0	0	0	0	0	0	0	0	0	0	0	0	0	0	0	0	0	0	0
Lawsonia intracellularis	0	0	0	0	0	0	0	0	0	0	0	0	0	0	0	0	0	0	0	0	0
Photorhabdus asymbiotica	0	0	0	0	0	0	0	0	0	0	0	0	0	1	0	1	1	0	0	0	0
Proteus mirabilis	0	0	0	0	0	0	0	0	0	0	0	0	0	0	0	0	1	0	0	0	0
Pseudomonas mendocina	0	0	0	0	0	0	0	0	0	0	0	0	0	0	0	0	0	0	0	0	0
Ramlibacter tataouinensis	0	0	0	0	0	0	0	0	0	0	0	0	0	0	0	0	0	0	0	0	0
Selenomonas sputigena	1	1	0	0	0	0	0	0	0	0	0	0	0	0	0	0	0	0	0	0	0
Shewanella violacea	0	0	0	0	0	0	0	0	0	0	0	0	0	0	0	0	0	0	0	0	0
Simkania negevensis	0	0	0	0	0	0	0	0	0	0	0	0	0	0	0	0	0	0	0	0	0

Sodalis glossinidius	0	0	0	0	0	0	0	0	0	0	1	0	0	1	0	0	0	0	0	0	0
Variovorax paradoxus	0	0	0	0	0	0	0	0	0	0	0	0	0	0	0	0	0	0	0	0	0
Vibrio Ex25	0	0	0	0	0	0	0	0	0	0	0	0	0	0	0	0	0	0	0	0	0
Vibrio harveyi	0	0	0	0	0	0	0	0	0	0	0	0	0	0	0	1	0	0	0	0	0
**effector**	**Tir**	**VP1680**	**VP1683**	**VP1686**	**VP1698**	**VirA**	**VopA**	**VopF**	**VopS**	**XopD**	**YopE**	**YopH**	**YopJ**	**YopK**	**YopO**	**YopQ**	**YopT**	**cp0034**	**ibe**	**Orf31**	**vpa0450**
Pseudomonas syringae	0	0	0	0	0	0	0	0	0	0	0	0	0	0	0	0	0	0	0	0	0
Acidovorax avenae	0	0	0	0	0	0	1	0	0	1	0	0	1	0	0	0	0	0	0	0	0
Acidovorax citrulli	0	0	0	0	0	0	1	0	0	1	0	0	1	0	0	0	0	0	0	0	0
Erwinia amylovora	0	0	0	0	0	0	0	0	0	0	0	1	0	0	0	0	0	0	0	0	0
Erwinia pyrifoliae	0	0	0	0	0	0	1	0	0	0	0	1	0	0	0	0	0	0	0	0	0
Ralstonia solanacearum	0	0	0	0	0	0	1	0	0	0	0	0	1	0	0	0	0	0	0	0	0
Sinorhizobium fredii	0	0	0	0	0	0	0	0	0	0	0	0	0	0	0	0	0	0	0	0	0
Xanthomonas campestris	0	0	0	0	0	0	1	0	0	1	0	0	1	0	1	0	0	0	0	0	0
Bradyrhizobium japonicum	0	0	0	0	0	0	0	0	0	0	0	0	0	0	0	0	0	0	0	0	0
Dickeya dadantii	0	0	0	0	0	0	0	0	0	0	0	0	0	0	0	0	0	0	0	0	0
Dickeya zeae	0	0	0	0	0	0	0	0	0	0	0	0	0	0	0	0	0	0	0	0	0
Erwinia tasmaniensis	0	0	0	0	0	0	0	0	0	0	0	1	0	0	0	0	0	0	0	0	0
Herbaspirillum seropedicae	0	0	0	0	0	0	0	0	0	0	0	0	0	0	0	0	0	0	0	0	0
Mesorhizobium loti	0	0	0	0	0	0	0	0	0	1	0	0	0	0	0	0	0	0	0	0	0
Pectobacterium atrosepticum	0	0	0	0	0	0	0	0	0	0	0	0	0	0	0	0	0	0	0	0	0
Pectobacterium carotovorum	0	0	0	0	0	0	0	0	0	0	0	0	0	0	0	0	0	0	0	0	0
Pseudomonas brassicacearum	0	0	0	0	0	0	0	0	0	0	0	0	0	0	0	0	0	0	0	0	0
Pseudomonas fluorescens	0	0	0	0	0	0	0	0	0	0	0	0	0	0	0	0	0	0	0	0	0
Rhizobium etli	0	0	0	0	0	0	0	0	0	1	0	0	0	0	0	0	0	0	0	0	0
Xanthomonas axonopodis	0	0	0	0	0	0	0	0	0	0	0	0	0	0	1	0	0	0	0	0	0
Xanthomonas oryzae	0	0	0	0	0	0	0	0	0	0	0	0	0	0	0	0	0	0	0	0	0
Salmonella bongori	0	0	0	0	0	0	1	0	0	0	1	0	1	0	0	0	0	0	1	0	0
Salmonella enterica	0	0	0	0	0	0	1	0	0	0	1	1	1	0	0	0	0	0	1	0	0
Yersinia enterocolitica	0	0	0	0	0	0	1	0	0	0	1	1	1	1	1	1	1	0	0	0	0
Yersinia pestis	0	0	0	0	0	0	1	0	0	0	1	1	1	1	1	1	1	0	0	0	0
Yersinia pseudotuberculosis	0	0	0	0	0	0	1	0	0	0	1	1	1	1	1	1	1	0	0	0	0

Burkholderia ambifaria	0	0	0	0	0	0	0	0	0	0	0	0	0	0	0	0	0	0	0	0	0
Burkholderia CCGE1001	0	0	0	0	0	0	0	0	0	0	0	0	0	0	0	0	0	0	0	0	0
Burkholderia CCGE1003	0	0	0	0	0	0	0	0	0	0	0	0	0	0	0	0	0	0	0	0	0
Burkholderia cenocepacia	0	0	0	0	0	0	0	0	0	0	0	0	0	0	0	0	0	0	0	0	0
Burkholderia gladioli	0	0	0	0	0	0	0	0	0	0	0	0	0	0	0	0	0	0	0	0	0
Burkholderia glumae	0	0	0	0	0	0	0	0	0	0	0	0	0	0	0	0	0	0	0	0	0
Burkholderia mallei	0	0	0	0	0	0	0	0	0	0	0	0	0	0	0	0	0	0	0	0	0
Burkholderia multivorans	0	0	0	0	0	0	0	0	0	0	0	0	0	0	0	0	0	0	0	0	0
Burkholderia pseudomallei	0	0	0	0	0	0	0	0	0	0	0	0	0	0	0	0	0	0	0	0	0
Burkholderia rhizoxinica	0	0	0	0	0	0	0	0	0	0	0	0	0	0	0	0	0	0	0	0	0
Burkholderia thailandensis	0	0	0	0	0	0	0	0	0	0	0	0	0	0	0	0	0	0	0	0	0
Burkholderia vietnamiensis	0	0	0	0	0	0	0	0	0	0	0	0	0	0	0	0	0	0	0	0	0
Chlamydophila abortus	0	0	0	0	0	0	0	0	0	0	0	0	0	0	0	0	0	1	0	0	0
Chlamydophila caviae	0	0	0	0	0	0	0	0	0	0	0	0	0	0	0	0	0	1	0	0	0
Chlamydophila felis	0	0	0	0	0	0	0	0	0	0	0	0	0	0	0	0	0	1	0	0	0
Chlamydophila pecorum	0	0	0	0	0	0	0	0	0	0	0	0	0	0	0	0	0	1	0	0	0
Chlamydophila pneumoniae	0	0	0	0	0	0	0	0	0	0	0	0	0	0	0	0	0	1	0	0	0
Chlamydophila psittaci	0	0	0	0	0	0	0	0	0	0	0	0	0	0	0	0	0	1	0	0	0
Pseudomonas aeruginosa	0	0	0	0	0	0	0	0	0	0	0	0	0	0	0	0	0	0	0	0	0
Shigella boydii	0	0	0	0	0	1	0	0	0	0	0	0	0	0	0	0	0	0	1	1	0
Shigella dysenteriae	0	0	0	0	0	1	0	0	0	0	0	0	0	0	0	0	0	0	1	1	0
Shigella flexneri	0	0	0	0	0	1	0	0	0	0	0	0	0	0	0	0	0	0	1	1	0
Shigella sonnei	0	0	0	0	0	1	0	0	0	0	0	0	0	0	0	0	0	0	1	1	0
Photorhabdus luminescens	0	0	1	0	0	0	0	0	0	0	0	0	0	0	0	0	1	0	0	0	1
Edwardsiella ictaluri	0	0	0	0	0	0	0	0	0	0	0	0	0	0	0	0	0	0	0	0	0
Shewanella baltica	0	0	0	0	0	0	0	0	0	0	0	0	0	0	0	0	0	0	0	0	0
Waddlia chondrophila	0	0	0	0	0	0	0	0	0	0	0	1	0	0	0	0	0	1	0	0	0
Parachlamydia acanthamoebae	0	0	0	0	0	0	0	0	0	0	0	0	0	0	0	0	0	1	0	0	0
Escherichia coli	1	0	0	0	0	1	0	0	0	0	0	0	0	0	0	0	0	0	1	1	0
Marinomonas mediterranea	0	0	0	0	0	0	1	0	0	0	0	0	1	0	0	0	0	0	0	0	0
Vibrio parahaemolyticus	0	1	1	1	1	0	1	1	1	0	0	0	1	0	0	0	0	0	1	0	1
Aeromonas salmonicida	0	0	1	1	0	0	0	0	1	0	1	1	0	0	1	0	0	0	0	0	1

Bordetella bronchiseptica	0	0	0	0	0	0	0	0	0	0	0	0	0	0	0	0	0	0	0	0	0
Bordetella parapertussis	0	0	0	0	0	0	0	0	0	0	0	0	0	0	0	0	0	0	0	0	0
Bordetella pertussis	0	0	0	0	0	0	0	0	0	0	0	0	0	0	0	0	0	0	0	0	0
Candidatus Hamiltonella	0	0	0	0	0	0	0	0	0	0	0	1	0	0	0	0	1	0	1	0	0
Chromobacterium violaceum	0	0	0	0	0	0	0	0	0	0	0	1	0	0	0	0	0	0	0	0	0
Citrobacter rodentium	1	0	0	0	0	1	0	0	0	0	0	0	0	0	0	0	0	0	1	1	0
Cupriavidus taiwanensis	0	0	0	0	0	0	0	0	0	0	0	0	0	0	0	0	0	0	0	0	0
Desulfovibrio vulgaris	0	0	0	0	0	0	0	0	0	0	0	0	0	0	0	0	0	0	0	0	0
Edwardsiella tarda	0	0	0	0	0	0	0	0	0	0	0	0	0	0	0	0	0	0	0	0	0
Geobacter bemidjiensis	0	0	0	0	0	0	0	0	0	0	0	0	0	0	0	0	0	0	0	0	0
Geobacter M21	0	0	0	0	0	0	0	0	0	0	0	0	0	0	0	0	0	0	0	0	0
Geobacter metallireducens	0	0	0	0	0	0	0	0	0	0	0	0	0	0	0	0	0	0	0	0	0
Hahella chejuensis	0	0	0	0	0	0	0	0	0	0	0	0	0	0	0	0	1	0	0	0	0
Hyphomicrobium MC1	0	0	0	0	0	0	0	0	0	0	0	0	0	0	0	0	0	0	0	0	0
Lawsonia intracellularis	0	0	0	0	0	0	0	0	0	0	0	0	0	0	0	0	0	0	0	0	0
Photorhabdus asymbiotica	0	0	0	1	0	0	0	0	1	0	0	0	0	0	0	0	1	0	0	0	0
Proteus mirabilis	0	0	0	0	0	0	0	0	0	0	0	1	0	0	0	0	0	0	0	0	0
Pseudomonas mendocina	0	0	0	0	0	0	0	0	0	0	0	0	0	0	0	0	0	0	0	0	0
Ramlibacter tataouinensis	0	0	0	0	0	0	0	0	0	0	0	0	0	0	0	0	0	0	0	0	0
Selenomonas sputigena	0	0	0	0	0	0	0	0	0	0	0	0	0	0	0	0	0	0	0	0	0
Shewanella violacea	0	0	0	0	0	0	0	0	0	0	0	0	0	0	0	0	0	0	0	0	0
Simkania negevensis	0	0	0	0	0	0	0	0	0	0	0	0	0	0	0	0	0	1	0	0	0
Sodalis glossinidius	0	0	0	0	0	0	0	0	0	0	0	0	0	0	0	0	0	0	0	0	0
Variovorax paradoxus	0	0	0	0	0	0	0	0	0	0	0	0	0	0	0	0	0	0	0	0	0
Vibrio Ex25	0	0	0	0	0	0	0	0	0	0	0	0	0	0	0	0	0	0	0	0	0
Vibrio harveyi	0	1	0	1	1	0	0	0	1	0	0	0	0	0	0	0	1	0	0	0	0

Supplementary table 4

	Occurrence			Occurrence	
Effector	Species	Genera	Effector	Species	Genera
PipB	40 (93)	23	YopE	6	3
SspH1	21	13	YopO	8 (10)	3
SlrP	24 (17)	11	AvrPphB	5	4
IpaB	15	6	ExoS	8	5
SipB	15	8	GALA	5 (3)	3
SopA	15	7	IpaH	5	2
AvrBsT	14	9	LscZ	5	4
YopH	12	8	SopD	6	4
VopA	12	8	XopD	6	4
YopJ	11	7 (3)	IncB	4	1
OspG	10	6	VP1686	5	3
SptP	12	6	VopS	4	3
SseJ	11	8(1)	BB4228	4	1
AvrA	10	5(3)	BPSS1516	3	2
ExoY	9	6	BPSS1539	3	1
OspF	10	5	ExoU	3	1
Cp0034	10	4	NleB	3	3
Ibe	10	6	VP1683	4	3
BopA	10	3	YopK	3	1
ExoT	8	6	YopQ	3	1
IpgD	9	4	vpa0450	3	3
LopT	8	5	EspB	2	2
OspC3	8	5	EspD	2	2
SopB	9	4	EspF	2	2
YopT	8	5	EspH	2	2
HopptoD2	7	7 (1)	EspZ	2	2
HopptoN	7	6 (1)	SifA	2	2
IcsB	7	2	SpiC	2	2
IpaA	7	3	Tir	2	2
IpaC	7	3	VP1680	3	1
SipA	7	3	VP1698	3	1
SipC	7	3	AexT	4	2
VirA	6	3	AvrPtoB		
orf31	7	3	IncA		
BopE	6	3	IncD		
EspG	6	3	IncE		
Ipgb	6	3	IncF		
MAP	6	3	IncG		
PopP1	9	5 (1)	SseF		
SopE	6	3	SseG		
SseL	6	4	TccP2		
TarP	6	1	VopF		
